# Binding free energy predictions of farnesoid X receptor (FXR) agonists using a linear interaction energy (LIE) approach with reliability estimation: application to the D3R Grand Challenge 2

**DOI:** 10.1007/s10822-017-0055-0

**Published:** 2017-09-09

**Authors:** Eko Aditya Rifai, Marc van Dijk, Nico P. E. Vermeulen, Daan P. Geerke

**Affiliations:** AIMMS Division of Molecular Toxicology, Department of Chemistry and Pharmaceutical Sciences, Vrije Universiteit Amsterdam, De Boelelaan 1108, 1081 HZ Amsterdam, The Netherlands

**Keywords:** Binding free energy prediction, Linear interaction energy, Applicability domain, Reliability estimation, Drug Design Data Resource, D3R Grand Challenge 2

## Abstract

Computational protein binding affinity prediction can play an important role in drug research but performing efficient and accurate binding free energy calculations is still challenging. In the context of phase 2 of the Drug Design Data Resource (D3R) Grand Challenge 2 we used our automated *eTOX ALLIES* approach to apply the (iterative) linear interaction energy (LIE) method and we evaluated its performance in predicting binding affinities for farnesoid X receptor (FXR) agonists. Efficiency was obtained by our pre-calibrated LIE models and molecular dynamics (MD) simulations at the nanosecond scale, while predictive accuracy was obtained for a small subset of compounds. Using our recently introduced reliability estimation metrics, we could classify predictions with higher confidence by featuring an applicability domain (AD) analysis in combination with protein–ligand interaction profiling. The outcomes of and agreement between our AD and interaction-profile analyses to distinguish and rationalize the performance of our predictions highlighted the relevance of sufficiently exploring protein–ligand interactions during training and it demonstrated the possibility to quantitatively and efficiently evaluate if this is achieved by using simulation data only.

**Electronic supplementary material:**

The online version of this article (doi:10.1007/s10822-017-0055-0) contains supplementary material, which is available to authorized users.

## Introduction

Drug development starts with the discovery of molecules that specifically and favorably interact with the pharmacological or drug target. In silico ligand-binding free energy (∆*G*_*bind*_) prediction is a valuable method in early drug discovery, as it can save resources by predicting (optimized) interactions between lead compounds and drug targets and/or off-target receptors [1, 2]. However, it is challenging to develop an accurate in silico prediction method that can be considered a descent trade-off with speed and efficiency [1–4]. To evaluate available methods for *∆G*_*bind*_ predictions and to get free-energy specialists further engaged in the development of the computer-aided drug discovery field, Drug Design Data Resource (D3R) held the Grand Challenge 2 (GC2, https://drugdesigndata.org/about/grand-challenge-2), a community challenge to predict binding poses and binding free energies of ligands without any affinity data provided to participants a priori. This blind prediction is invaluable as unbiased test for current state-of-the-art methods and can serve as catalyst for further development. In GC2, the challenge is to predict binding free energies of agonists of farnesoid X receptor (FXR), a protein belonging to the nuclear receptor superfamily and is mainly expressed in liver, intestine, adrenal gland, and kidney. FXR is known to play a key role in regulating cholesterol and bile acid homeostasis, hence FXR agonists can be potential therapeutics for dyslipidemia and diabetes [5].

There is a variety of methods for calculating *∆G*_*bind*_, from empirical scoring functions to more reliable and robust alchemical free energy methods such as thermodynamic integration (TI) [6] and free energy perturbation (FEP) [7]. As an alternative, end-point methods such as molecular mechanics combined with Poisson–Boltzmann or generalized Born and surface area continuum solvation (MM/PBSA or MM/GBSA) [8] and linear interaction energy (LIE) [9] perform faster than alchemical free energy methods but can still be more accurate compared to empirical scoring functions by explicitly including protein- and ligand-conformational sampling. In LIE, binding free energies are directly calculated from differences in ligand–surrounding interaction energies between the bound and unbound states, without including solvent–access and entropic terms as in MM/PSBA [2], but LIE predictions need pre-calibration of empirical parameters for the system of interest based on an external set of training data [10].

Here we use our automated implementation [11, 12 and L. Capoferri et al. (submitted)] of an iterative version of LIE [13] in which protein–ligand binding poses (as obtained from ligand docking into possible different protein conformations [14]) are used as input for different molecular dynamics (MD) simulations of the bound state. The predicted free energy of binding ($\Delta {G_{pred}}$) is estimated as the weighted sum of MD ensemble-averaged electrostatic ($\Delta V_{i}^{{ele}}$) and van der Waals ($\Delta V_{i}^{{vdw}}$) interaction energies between the ligand (*lig*) and its surrounding (*surr*) in complex with the protein (*bound*) and free in solution (*unbound*) by following linear response theory (Eq. 1).

1 $$\Delta {G_{pred}}=~\alpha \mathop \sum \limits_{i}^{N} {W_i}\Delta V_{i}^{{vdw}}+~\beta \mathop \sum \limits_{i}^{N} {W_i}\Delta V_{i}^{{ele}}+~\gamma$$

$\Delta V_{i}^{{vdw}}$ and $\Delta V_{i}^{{ele}}$ are equal to $\left\langle {V_{{lig - surr\,}}^{{vdw}} } \right\rangle _{{bound,i}} - ~\left\langle {V_{{lig - surr\,}}^{{vdw}} } \right\rangle _{{unbound}}$ and $\left\langle {V_{{lig - surr\,}}^{{ele}} } \right\rangle _{{bound,i}} - ~\left\langle {V_{{lig - surr\,}}^{{ele}} } \right\rangle _{{unbound}}$ as obtained for the independent simulations *i* of the bound complex. LIE parameters *α* and *β* are empirically calibrated and the off-set parameter *γ* can be optionally included in the equation. The contributions of each individual simulation are calculated by weighting them as follows [13]:

2 $${W_i}=~\frac{{{e^{ - \Delta {G_{pred,i}}/{k_B}T}}}}{{\mathop \sum \nolimits_{i} {e^{ - \Delta {G_{pred,i}}/{k_B}T}}}}$$

The need for pre-calibrated parameters poses challenges on the availability of sufficient data for training, and may lead to limited applicability of a trained model in terms of the chemical space covered by the training compounds. Here we used experimentally observed binding free energies ($\Delta {G_{obs}}$) [5, 15–17] for the different D3R GC2 subclasses of FXR binders to develop three different local LIE models for the subclasses. In addition, we used our recently introduced approach for quantitative applicability domain (AD) analysis [12] to evaluate if chemical space was sufficiently covered by the training compounds used for model calibration. Several methods to quantitatively perform AD analyses have been reported for ligand-based QSAR approaches before [18, 19]. However, this task is especially challenging when information on protein structure, interactions and/or dynamics is included as well in the prediction, like in LIE modeling. As a remedy, our AD approach [12] evaluates the applicability and reliability of a given LIE model towards (sets of) query compounds based on simulation data only. We previously tested the performance of our method for AD analysis to a structurally diverse set of binders of the flexible cytochrome P450 isoform 1A2 (CYP 1A2) and could successfully distinct a multifarious subset of 14 external test compounds with experimental accuracy in their binding free-energy prediction, from eight outliers in the test set [12]. In the current study, we evaluate our fully automated pipeline [L. Capoferri et al. (submitted)] for training and reliability assessment of iterative LIE models for the prediction of FXR binding free energies for D3R GC2 compounds. The pipeline requires prepared protein and ligand structures as only input, and uses MD simulations on the ns time scale only.

## Computational methods

### Model training for the D3R Grand Challenge 2 dataset

For GC2, we collected data sets of experimentally estimated FXR-binding IC_50_ values from literature via ChEMBL [20], which were used to obtain the Cheng-Prusoff estimate of observed binding free energies $\Delta {G_{obs}}$ [21]. These ∆*G*_*obs*_ values were used for training local LIE models for the benzimidazole [5, 15] [IC_50_ assay method: Scintillation proximity assay (SPA)], isoxazole [16] (SPA), and sulfonamide [17] [time-resolved fluorescence energy transfer (TR-FRET)] classes of compounds. The external data sets for the benzimidazoles and sulfonamides were split into a literature training and test set, which are presented in Tables S1 and S2 of the supplementary material together with $\Delta {G_{obs}}$ values and ChEMBL identifiers. Molecular structures of the training and test set compounds can be found in Figures S1 and S2 of the supplementary material. The models were subsequently used for $\Delta {G_{pred}}$ predictions for the respective classes of D3R compounds (Figure S3); for the D3R spiro-containing compounds, the sulfonamide model was used.

**Fig. 1:**
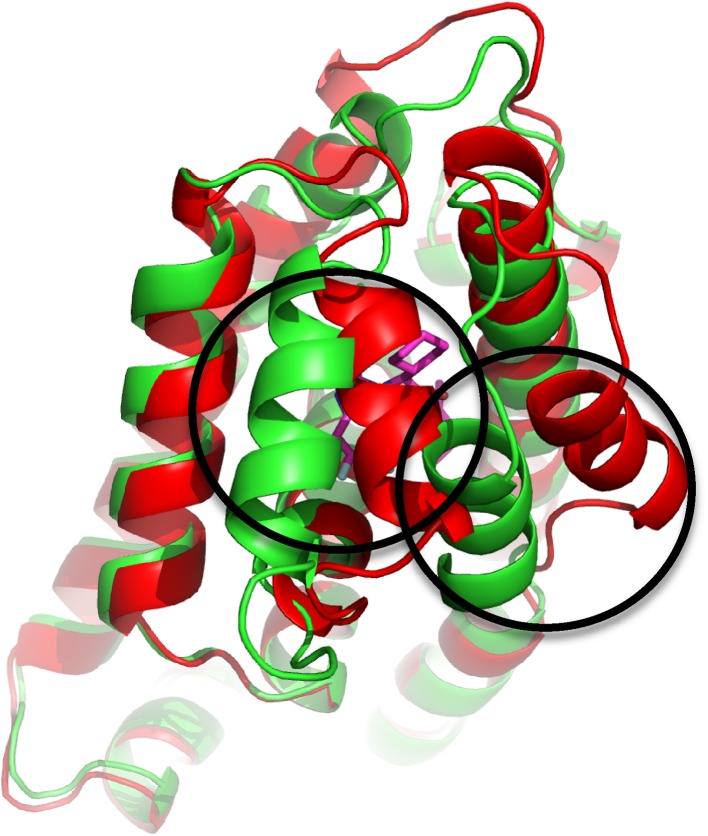
Illustration of the observed variation of FXR crystal structure conformations in terms of the helices adjacent to the co-crystallized binding site of ligand (conformation 1: *green* (PDB ID 3OMK [15]), conformation 2: *red* (3FXV [16])). Figure was generated using PyMOL (The PyMOL Molecular Graphics System, version 1.8 Schrödinger, LLC.)

For the selection of protein crystal structures for use in docking and subsequent MD, we found that crystal structures provided by Roche at the start of the second stage of GC2 (https://drugdesigndata.org/about/grand-challenge-2) can be grouped in two types of structures (conformation 1 or 2, Fig. 1), based on the conformation of the helices adjacent to the binding site of the co-crystallized ligands. This observation is in line with comparisons to the FXR structures from PDB [5, 15, 16]. Based on the protein conformations observed in the co-crystallized structures of Roche, we chose to use PDB structure 3OMK [15] (conformation 1) as protein template for use in the benzimidazole LIE model, and conformation 2 structures as templates for the isoxazole (3FXV) [16] and sulfonamide models (3BEJ) [22]. For the miscellaneous compounds in the D3R data set, the benzimidazole, isoxazole, or sulfonamide model was used to calculate $\Delta {G_{pred}}$, depending on the protein conformation obtained for the miscellaneous ligand in the crystal structure provided by Roche. Protein structure preparation steps before docking (addition of missing atoms and residues, assignment and fixing of charged and protonation states, deletion of atoms with fractional occupancies, and subsequent energy minimization) were conducted using ModLoop [23] and UCSF Chimera version 1.10.2 [24].

### Binding free energy prediction workflow

For model training and testing and for the predictions submitted to GC2, we used our in-house pipeline *eTOX ALLIES* [L. Capoferri et al. (submitted)], which works as an automated workflow to combine molecular docking, MD, and the iterative LIE method, in order to calculate $\Delta {G_{bind}}$ of target compounds. It uses automated least-square fitting to train model parameters based on the curated experimental binding free energies. The trained model can subsequently be used to predict $\Delta {G_{pred}}$ of query compounds, and the reliability of a prediction is indicated in terms of the cumulative score in confidence index (CI) values obtained from AD assessment, see below.

**Fig. 2:**
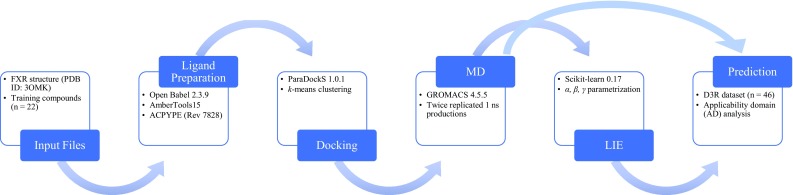
Schematic overview of the automated (iterative) LIE workflow used in this work. The pipeline starts from ligand and protein structure preparation, followed by docking and MD simulations. The LIE parameters are trained based on MD trajectories and used for predicting $\Delta {G_{bind}}$ for external test or D3R compounds

After the stereochemistry of training or query compounds was inspected and corrected in 3D format using MOE (MOE version 2015.10, Chemical Computing Group Inc., Canada), their 3D SMILES string was used as input for *eTOX ALLIES*, which uses Open Babel 2.3.9 [25] to perform ligand preparation (generation of 3D coordinates if necessary and neutralization or protonation according to pH 7.4 depending on the model settings). All compounds from the literature training and test sets were simulated in neutral forms. AmberTools15 [26] was then employed to create ligand topologies for use in MD according to the General Amber Force Field (GAFF) [27] and AM1-BCC QM potential [28], and this full topology generation was run automatically and converted to GROMACS format using ACPYPE (Rev: 7828) [29]. Docking was carried out by ParaDockS 1.0.1 [30] with a docking radius of 1.0 nm and the docking center set to the center-of-mass coordinates of co-crystallized ligands. Representative docked ligand conformations used as input structures for MD were obtained after principle component driven geometric clustering of the obtained ligand docking poses. For that purpose, principle component analysis (PCA) was performed on the docking poses with coordinates of the heavy atoms taken as the variables [12]. After dimensionality reduction, the PCA scores were used for *k*-means clustering [31]. Any additional component or cluster was taken into account if this led to a further increment of at least 5% of the explained variance in coordinate space or scores, respectively. The medoids of the obtained clusters were chosen as representative binding poses (typically 2–3 per ligand) and were used as input for the MD simulations. Prior to MD, protein–ligand structures were energy minimized, solvated in TIP3P water [32] molecules (~11,000), and Cl^−^ and Na^+^ counter ions were added to neutralize the system. Thermal pre-equilibration, temperature coupling, pressure coupling, (grid-based) pair-list update frequency, and long-range treatment of non-bonded interactions during MD were performed as described previously [12]. All energy minimizations and MD simulations including 1 ns production runs were performed using GROMACS 4.5.5 [33] and the Amber14SB force field to describe the protein [34]. Ordinary least squares (OLS) fitting for model training was performed using the Python scikit-learn 0.17 [35] package, and LIE parameters (i.e., *α, β*, and *γ*) from training (see below) were used to predict $\Delta {G_{pred}}$ of the D3R challenge compounds. An overview of the workflow is depicted in Fig. 2.

For every compound, *∆G*_*bind*_ was calculated using average ligand-environment interaction energies as obtained from the multiple MD simulations that started from the different poses obtained from clustering of binding poses during molecular docking [12], using weighting according to Eq. 2 [13], and simulations were run twice per binding pose [14] with interaction energy values written out to disk every 10 ps. Average interaction energies for unbound ligands were obtained from separate duplicated 1 ns production simulations of the ligand solvated in (approximately 650 mol) TIP3P water molecules [32] using the same MD settings as for the protein–ligand complexes. Subsequently, interaction profiles between ligands and FXR as obtained from the simulations for all binding poses were analyzed using an in-house Python script, to identify protein ligand interaction types using rule-based protocols described in the supplementary material of [36].

### AD assessment

As recently introduced by us [12], the reliability of the LIE predictions was estimated using the following quantitative AD assessment approach. We use five AD criteria or confidence indices (CIs) to obtain this estimation. The CIs include the four parameters described in reference [12], together with an estimate if $\Delta {G_{pred}}$ falls within the range of experimental values used in model training. A score of 0 is assigned per index according to the following rules:

1. $\Delta {G_{pred}}$ should fall within the range defined by the minimum and maximum values of calculated ∆*G*_*bind*_ of training compounds.
2. The chemical similarity of the ligand (represented as Tanimoto score, TS) should be higher than the cutoff defined, which is the lowest TS value gained by comparing each training compound with the most similar compound within the training set.
3. Average ligand–protein interaction energies in terms of the $\Delta V_{i}^{{vdw}}$ and $\Delta V_{i}^{{ele}}$ should fall within the 95 percentiles of the training compounds based on the Mahalanobis distance calculated from the centroid for the training compounds.
4. The per-residue decomposition to the van der Waals interaction energies of the test compounds are projected onto the principal component analysis (PCA) space of the training compounds score as well as the orthogonal distances, and should be within the 95 percentiles of the training compounds distribution.
5. Per-residue decomposition to the electrostatic interactions, evaluated in the same way as the van der Waals interaction energies under 4.

For every violation, the score for the corresponding rule is assigned a value of 1. The resulting value for the (total) CI score belonging to the binding free-energy prediction of a given query compound is then obtained by summation of the individual scores, and ranges from 0 (no violation and high confidence) until 5 (all violated and low confidence) [12].

## Results and discussion

### Model training and testing based on literature data

**Fig. 3:**
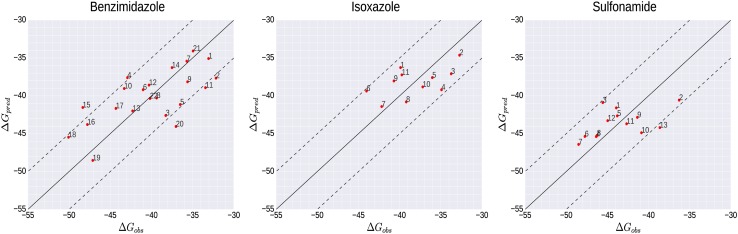
Correlations between experimentally observed and calculated binding free energies ∆*G* (kJ mol^−1^) for the literature training compounds in the benzimidazole, isoxazole, and sulfonamide models. The *solid line* represents ideal correlation and *dashed lines* indicate an error interval of ±5 kJ mol^−1^

The results of the training of our LIE models are summarized in Table 1 and S1 (supplementary material). Correlations between observed experimental $\Delta {G_{obs}}$ and calculated ∆*G*_*pred*_ values are depicted in Fig. 3. The performance of the models was evaluated by two types of quality metrics, i.e., in terms of correlation coefficients (r Pearson and *ρ* Spearman) and averaged deviations from experimental values (i.e., root-mean-square error for the training set (RMSE), and standard error in prediction (SDEP_LOO−CV_) using leave-one out cross-validation). The benzimidazole model showed higher correlation coefficients (r = 0.68, *ρ* = 0.65) compared to the isoxazole and sulfonamide models (r = 0.55, *ρ* = 0.52 and r = 0.52, *ρ* = 0.58, respectively), and higher values for LIE parameters *α* and *β* (and a lower off-set *γ* value). RMSE and SDEP_LOO−CV_ of the benzimidazole model are larger than for the two other models (Table 1), but still within typical experimental accuracy [37].

**Table 1:** Model parameters for the LIE models for the benzimidazole, isoxazole and sulfonamide classes of compounds, and respective errors and correlation metrics for the literature training sets

	Benzimidazole	Isoxazole	Sulfonamide
*α*	0.33	0.10	0.14
*β*	0.12	0.10	0.08
*γ*	−13.0	−31.8	−33.4
RMSE	3.8	2.9	2.9
SDEP_LOO-CV_	4.1	3.7	3.8
r Pearson	0.68	0.55	0.52
*ρ* Spearman	0.65	0.52	0.58

*γ*, root-mean-square error (RMSE) and standard error of prediction for leave-one our cross-validation (SDEP_LOO−CV_) are given in kJ mol^−1^

We tested the performance of the three models by computing binding free energies for the external test sets obtained from literature (Table 2). For the benzimidazole models, r Pearson is 0.64 but the standard error in prediction (SDEP_CV_) for the test set is relatively high (5.9 kJ mol^−1^). AD assessment was then applied to this dataset, and the compounds were categorized based on their CI scores (Table 2 and S2). From the 23 compounds of the benzimidazole test set, 8 compounds were categorized with CI score = 0, 6 showed a CI score of 1, and 9 compounds had a CI score of 2. By evaluating the deviation of $\Delta {G_{pred}}$ from experimental $\Delta {G_{obs}}$ per CI category (Table 3), it can be seen that for the compounds with predicted confidence score = 0, SDEP_CV_ is within experimental accuracy [37], whereas for increased CI scores this deviation is larger. This finding supports the use of our AD assessment approach to predict the quality of our predictions. It should be noted however that in this specific case the number of literature test compounds with CI score = 0 was relatively low (8/23).

**Table 2:** Overall statistics for literature test set for the benzimidazole and sulfonamide models, including standard errors of prediction (SDEP_CV_ in kJ mol^−1^), total number of literature test compounds (n), and number of compounds per CI category (with CI values representing the number of AD criteria violated by the respective subsets of ligands)

	n	SDEP_CV_	r Pearson	CI = 0	CI = 1	CI = 2	CI = 3	CI = 4	CI = 5
Benzimidazole	23	5.9	0.64	8	6	9	–	–	–
Isoxazole	–	–	–	–	–	–	–	–	–
Sulfonamide	7	3.9	−0.23	–	3	3	1	–	–

**Table 3:** Standard deviation in prediction (SDEP_CV_ in kJ mol^−1^) and correlation metrics for benzimidazole compounds of the literature test set for all test set benzimidazole predictions (All), and as specified per subsets of test compounds with indicated CI scores

	n	SDEP_CV_	r Pearson	*ρ* Spearman
All	23	5.9	0.64	0.56
CI = 0	8	5.0	0.79	0.71
CI = 1	6	6.8	0.59	0.71
CI = 2	9	6.1	0.61	0.37
CI = 3	0	–	–	–
CI = 4	0	–	–	–
CI = 5	0	–	–	–

The confidence in our predictions using the isoxazole and sulfonamide models was affected by limited availability of calibration data and the lack of test compounds with CI score = 0 (Table 2 and S2). The limited number of training data (n = 11) for isoxazoles hampered the use of an external test set and for the seven sulfonamide test compounds from literature, the linearity metrics showed negative correlation with experiment (r Pearson = −0.23) and no compound fell in the CI = 0 category despite the ligand similarity between training and test compounds. Ligand similarity was confirmed by our observation that the score for AD criteria 2 was 0 for all test compounds, indicating that similar compounds may be involved in different protein–ligand interactions during simulation.

### D3R compound predictions

The D3R Grand Challenge 2 involved predicting $\Delta {G_{pred}}$ for 102 FXR agonists. Here, we report the performance of our workflow for this set of compounds. The prediction workflow is identical to the ones used for calculating $\Delta {G_{pred}}$ values for the literature test set. We submitted predictions for 46 benzimidazole, 4 isoxazole, and 6 miscellaneous compounds. For the sulfonamide and spiro compounds, we only submitted compounds with CI score ≤ 2, including respectively 3 and 1 compounds only. The details on our D3R predictions can be found in Table S2 of the supplementary material and are summarized in Table 4.

**Table 4:** Total number of predictions (n) and number of predictions per CI category for each subclass model of the D3R dataset (with CI values representing the number of AD criteria violated by the respective ligands)

	n	CI = 0	CI = 1	CI = 2	CI = 3	CI = 4	CI = 5
Benzimidazole	46	9	19	15	2	1	–
Isoxazole	4	–	2	–	1	1	–
Sulfonamide	3	–	–	3	–	–	–
Spiro	1	–	–	1	–	–	–
Miscellaneous	6	–	–	3	1	2	–

After the experimentally determined binding affinities for the D3R compounds were released, it was inferred that the submitted sulfonamide, spiro, isoxazole and miscellaneous compound predictions showed substantial deviation from experimental values. SDEP values were 16.3, 8.3, 4.1 and 7.5 kJ mol^−1^ respectively, in line with CI values of 2 (or higher for the miscellaneous compounds), Table 4 and S2. The individual CI scores indicate a narrow scope of the diversity in structures and protein interactions covered by the corresponding training sets used, and/or a restricted range of $\Delta {G_{obs}}$ values e.g. for the sulfonamide training set (−36.3 to −48.5 kJ mol^−1^, Table S1) when compared to the ranges of $\Delta {G_{obs}}$ values of −24.8 to −38.0 kJ mol^−1^ and −24.8 to −45.2  kJ mol^−1^ for the D3R sulfonamide and spiro compounds, respectively, Table S2.

For the D3R benzimidazoles, 9 out of 46 compounds fell into the CI score = 0 category while the rest fell into CI = 1 until CI = 4 (Table 5 and S2). After the experimental values were released, we found that SDEP_CV_ is lowest and within 5.0 kJ mol^−1^ for the nine compounds with CI score = 0. However, when one or two of the AD criteria parameters was violated (for in total 34 ligands), the attributed SDEP_CV_ is substantially higher, i.e., 9.6 and 8.8 kJ mol^−1^, respectively, Table 5. For the compounds with either CI = 3 or CI = 4, the trend is less conclusive (with SDEP_CV_ values of 1.1 and 16.7 kJ mol^−1^, Table 5), but this refers to three compounds only and it does not change our finding that when considering binders with CI score = 0, the SDEP decrease to 5.0 kJ mol^−1^ and r Pearson and *ρ* Spearman increase up to 0.51 and 0.37, respectively, compared to the corresponding data for all predictions. Nevertheless, it should be realized that this includes a limited number of compounds and more diverse experimental calibration data would be needed to extend this dataset further.

**Table 5:** Standard deviation in prediction (SDEP_CV_ in kJ mol^−1^) and correlation metrics for benzimidazole compounds of the D3R set for all D3R benzimidazole predictions (All) and as specified per subset of compounds with indicated CI score

	n	SDEP_CV_	r Pearson	*ρ* Spearman
All	46	8.6	−0.09	−0.08
CI = 0	9	5.0	0.51	0.37
CI = 1	19	9.6	−0.29	−0.33
CI = 2	15	8.8	0.04	0.07
CI = 3	2	1.1	–	–
CI = 4	1	16.7	–	–
CI = 5	0	–	–	–

### Protein–ligand interaction profile analysis

**Fig. 4:**
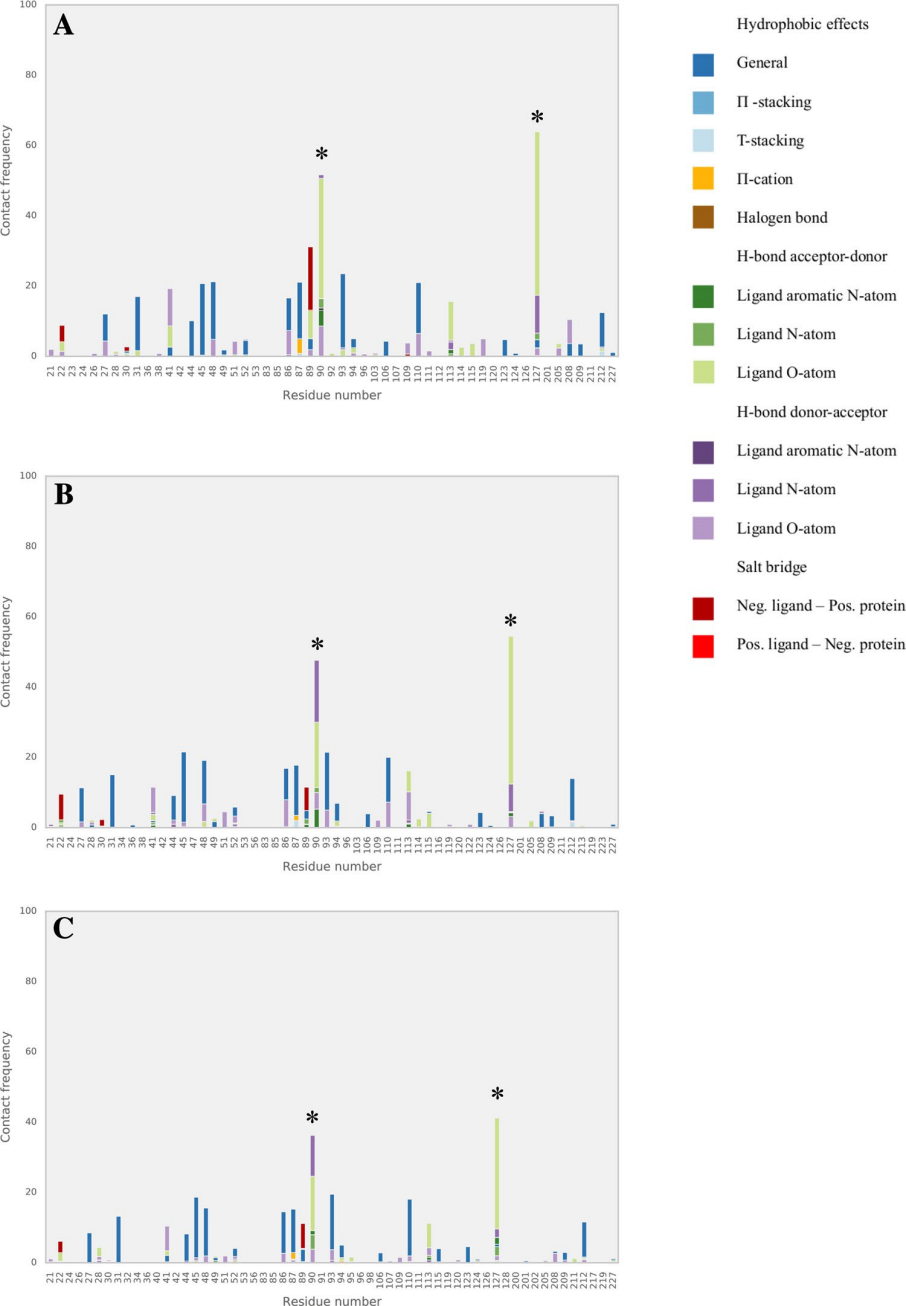
Relative frequencies of FXR interactions during MD with training (**a**) literature test (**b**) and D3R (**c**) sets of benzimidazole ligands. *Asterisks* indicate hotspots Ser90 and Thr127

**Fig. 5:**
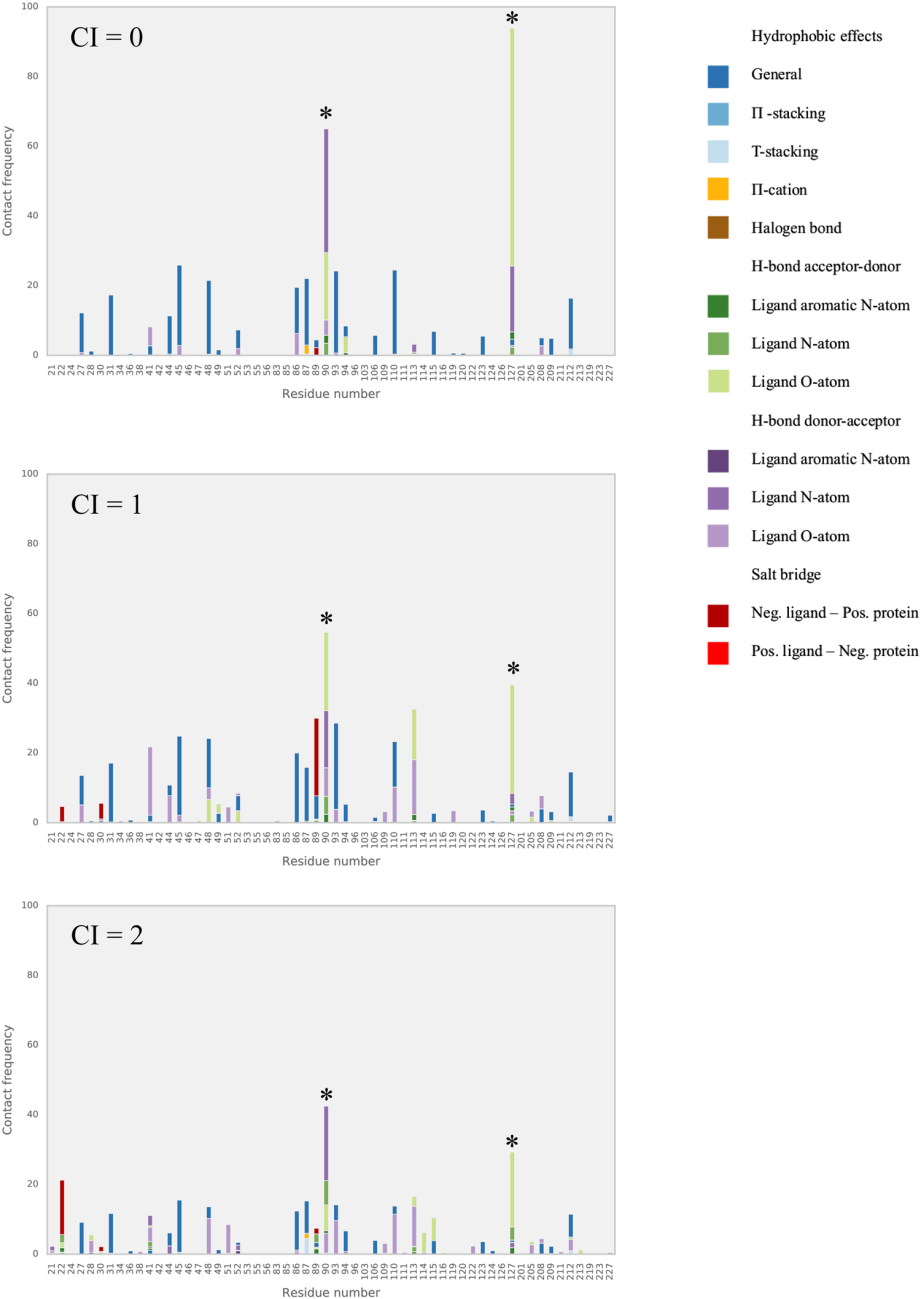
Relative frequencies of FXR interactions during MD with literature benzimidazole test compounds with a CI score of 0, 1, or 2. *Asterisks* indicate hotspots Ser90 and Thr127

**Fig. 6:**
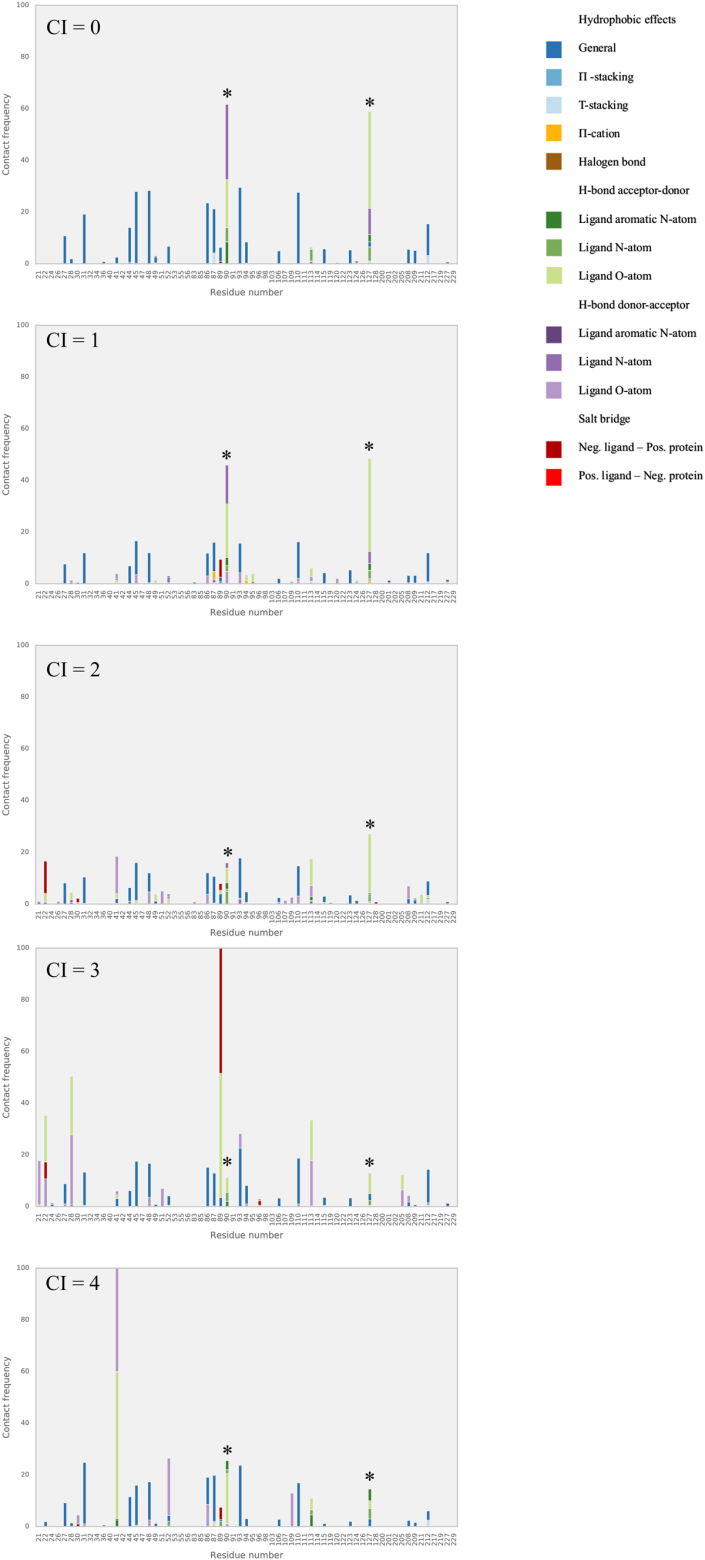
Relative frequencies of FXR interactions during MD with D3R benzimidazole compounds with a CI score of 0, 1, 2, 3, or 4. *Asterisks* indicate hotspots Ser90 and Thr127

We further analyzed our benzimidazole model in terms of the interactions with the protein target as observed during the MD simulations. We evaluated the dominant interactions between the benzimidazole compounds and amino acid residues of the FXR active site and checked whether they have consensus with each other as well as with the ones featured in available co-crystalized structures. The frequency with which each ligand–residue interaction occurs per simulation is described as horizontally stacked bar (distinguished by colors for different types of interactions) in Figs. 4, 5 and 6. Because of their relatively large abundancy, hydrophobic contact frequencies are presented in the figures after being divided by an arbitrary factor of 10.

**Fig. 7:**
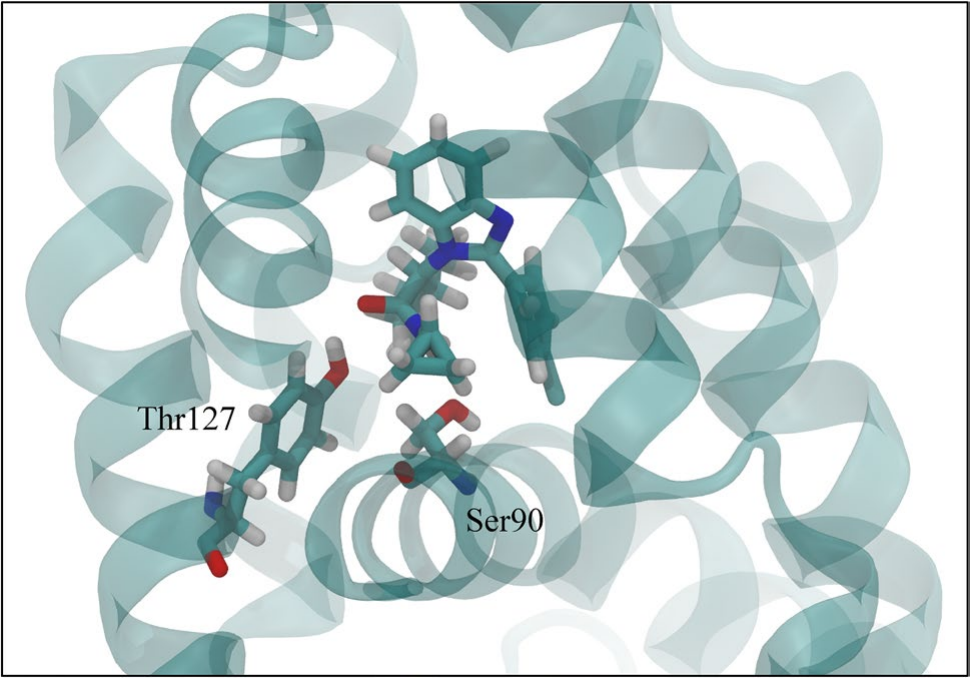
Training compound CHEMBL1642356 in complex with FXR, residues Ser90 and Thr127 are shown in *stick* representation as well. Figure was generated using VMD version 1.9.2 [38]

Figure 4 shows that in the simulations of the training, literature test and D3R compounds, hydrogen bonds frequently occur between ligands and two hotspots identified in the FXR structure, i.e., Ser90 and Thr127, see Fig. 7. These key interaction residues were also reported in literature studies of FXR crystal structures in complex with benzimidazole compounds [5, 15] and can be observed as well in the Roche crystal structures for the 21 FXR-benzimidazole complexes that were released during GC2.

When inspecting the interaction profiles obtained from simulations of test and D3R compounds as clustered based on the CI scores for their predictions, the profiles varied between them in a similar way for both subsets, Figs. 5 and 6. Our analysis shows that for the compounds for which our AD analysis predicts highest confidence in $\Delta {G_{pred}}$ (i.e. for those with CI score = 0), Ser90 and Thr127 interactions are most frequently observed. Figures 5 and 6 show that their frequencies become smaller for predictions with lower confidence (i.e., with higher CI score) and that hotspot interactions are in these cases taken over by interactions with other residues such as Arg89 and Asn41. This indicates that compounds with lower CI score (higher confidence) tend to have more similar dominant interactions, with a higher frequency and less mixed with interactions to other non-hotspot residues. Interestingly, the high frequency of hotspot interactions observed during MD of the D3R compounds with lowest CI score did not necessarily coincide with a correspondence between the starting ligand-binding poses for MD and binding orientations observed in crystal structures from Roche (as released during GC2). Whereas the crystal structures show large correspondence in typical binding poses for benzimidazole compounds, we observed root-mean-square deviations in atomic positions between docked MD-starting poses and corresponding poses in the Roche structures of up to 0.5 nm. When retrospectively calibrating an LIE model for selected benzimidazole compounds from the D3R dataset (with the benzimidazole co-crystallized Roche structure *1hoia* as protein template) for which we could use docked poses with similar binding orientations as in the co-crystallized D3R structures only, we obtained a benzimidazole LIE model with similar values for *α* (0.33), *β* (0.08) and *γ* (−13.0 kJ mol^−1^) but with lower predictivity (RMSE = 4.4 kJ mol^−1^, SDEP_LOO−CV_ = 4.8 kJ mol^−1^, r Pearson = 0.41, *ρ* Spearman = 0.28) than for our trained benzimidazole model, *cf*. Table 1.

## Conclusions

We reported here our participation in D3R GC2 by employing *eTOX ALLIES*, an automated workflow for binding affinity prediction with AD analysis. The methodology consists of molecular docking, MD simulation, and iterative LIE to calculate binding free energies of query compounds by using pre-calibrated parameters obtained from curated experimentally observed binding free energies from literature. This semi-empirical end-point method represents an alternative for calculating binding affinities with feasible speed and accuracy.

When applied to the blind binding affinity prediction for D3R GC2 farnesoid X receptor agonists, we assessed the predictive reliability by attributing applicability domain (AD) analyses to measure the confidence of the performed calculations. Using confidence scores we were able to distinguish predictions with low and high confidence, which can thus be an indicator of the performance of our approach. For the small set of (9) predictions with highest confidence, experimental accuracy was obtained (SDEP = 5.0 kJ mol^−1^). We additionally analyzed protein–ligand interactions during MD and compared MD-starting ligand-binding poses with crystal structure data, and observed that the frequency of hotspot interactions (with FXR residues Ser90 and Thr127) gave a direct indication of the confidence in our predictions.

## Electronic supplementary material

Below is the link to the electronic supplementary material.

Supplementary material 1 (PDF 268 KB)

## Abbreviations

AD: Applicability domain
CI: Confidence index
D3R: Drug Design Data Resource
FEP: Free energy perturbation
FXR: Farnesoid X receptor
GC2: Grand Challenge 2
LIE: Linear interaction energy
MD: Molecular dynamics
QSAR: Quantitative structure–activity relationship
RMSE: Root-mean-square error
SDEP: Standard deviation in prediction
TI: Thermodynamic integration
