# Supplementary material for: Binding free energy predictions of farnesoid X receptor (FXR) agonists using a linear interaction energy (LIE) approach with reliability estimation: application to the D3R Grand Challenge 2
Source: J Comput Aided Mol Des. 2017 Sep 9;32(1):239–49. doi: 10.1007/s10822-017-0055-0 (PMC5767202; doi:10.1007/s10822-017-0055-0)
Supplement: Supplementary file 1 — Supplementary material 1 (PDF 268 KB) [file 10822_2017_55_MOESM1_ESM.pdf]

SUPPLEMENTARY MATERIAL of the manuscript

Binding free energy predictions of Farnesoid X receptor (FXR) agonists using a Linear Interaction Energy (LIE) approach with reliability estimation: application to the D3R Grand Challenge 2

by: Eko Aditya Rifai, Marc van Dijk, Nico P.E. Vermeulen, and Daan P. Geerke

Table S1. Dataset of 46 FXR ligands obtained from literature for training of LIE models for the benzimidazole, isoxazole and sulfonamide classes of compounds. Listed are the ligand ID used throughout the current work; ChEMBL IDs of the ligands; literature references (Ref.) from which IC50 values were retrieved to obtain the experimentally observed FXR binding free energy ΔG_{obs} (in kJ mol^{-1}), calculated as ΔG_{obs} = RT ln (IC_{50}/2); the predicted binding free energy (ΔG_{pred}) in kJ mol^{-1}; and differences $\Delta G_{obs} - \Delta G_{pred}$ (Diff., in kJ mol^{-1}).

Compound class	ID	ChEMBL ID	Ref.	ΔG_{obs}	ΔG_{pred}	Diff.
Benzimidazoles	1	CHEMBL1642356	[5]	-33.1	-35.1	2.0
	2	CHEMBL1642359	[5]	-32.2	-37.7	5.5
	3	CHEMBL1642358	[5]	-38.3	-42.6	4.3
	4	CHEMBL1642346	[5]	-42.9	-37.6	-5.3
	5	CHEMBL1642350	[5]	-36.5	-41.1	4.6
	6	CHEMBL1642354	[5]	-41.0	-39.2	-1.8
	7	CHEMBL1642347	[5]	-35.7	-35.4	-0.3
	8	CHEMBL1668262	[15]	-39.4	-40.3	0.9
	9	CHEMBL1668242	[15]	-35.6	-38.1	2.5
	10	CHEMBL1668247	[15]	-43.3	-39.0	-4.3
	11	CHEMBL1668256	[15]	-33.4	-38.9	5.5

	12	CHEMBL1668254	[15]	-40.3	-38.6	-1.7
	13	CHEMBL1668253	[15]	-42.3	-42.0	-0.3
	14	CHEMBL1668240	[15]	-37.5	-36.3	-1.2
	15	CHEMBL1668244	[15]	-48.3	-41.5	-6.8
	16	CHEMBL1668259	[15]	-47.8	-43.8	-4.0
	17	CHEMBL1668258	[15]	-44.3	-41.7	-2.7
	18	CHEMBL1615154	[15]	-50.1	-45.5	-4.6
	19	CHEMBL1668248	[15]	-47.1	-48.5	1.4
	20	CHEMBL1668245	[15]	-37.0	-44.1	7.0
	21	CHEMBL1668260	[15]	-35.0	-34.1	-0.9
	22	CHEMBL1668261	[15]	-40.2	-40.3	0.2
Isoxazoles	1	CHEMBL511389	[16]	-39.9	-36.3	-3.6
	2	CHEMBL511559	[16]	-32.7	-34.6	1.9
	3	CHEMBL466880	[16]	-33.7	-37.1	3.3
	4	CHEMBL466471	[16]	-34.9	-39.2	4.3
	5	CHEMBL513149	[16]	-36.0	-37.6	1.6
	6	CHEMBL466523	[16]	-44.0	-39.4	-4.7
	7	CHEMBL466323	[16]	-42.2	-41.4	-0.8
	8	CHEMBL466887	[16]	-39.2	-40.8	1.6
	9	CHEMBL466318	[16]	-40.7	-38.0	-2.7
	10	CHEMBL469135	[16]	-37.2	-38.8	1.6
	11	CHEMBL466315	[16]	-39.7	-37.2	-2.5
Sulfonamides	1	CHEMBL1367639	[17]	-43.9	-41.6	-2.3
	2	CHEMBL3264627	[17]	-36.3	-40.6	4.3
	3	CHEMBL3264635	[17]	-45.6	-40.9	-4.7
	4	CHEMBL3264639	[17]	-46.4	-45.4	-1.0
	5	CHEMBL3264640	[17]	-43.8	-42.6	-1.2
	6	CHEMBL3264642	[17]	-47.7	-45.4	-2.4
	7	CHEMBL3264644	[17]	-48.5	-46.4	-2.1
	8	CHEMBL3264645	[17]	-46.3	-45.2	-1.0
	9	CHEMBL3264646	[17]	-41.4	-42.8	1.5
	10	CHEMBL3264648	[17]	-40.9	-44.9	4.0
	11	CHEMBL3264650	[17]	-42.7	-43.7	1.0
	12	CHEMBL3264651	[17]	-45.0	-43.3	-1.7
	13	CHEMBL3264987	[17]	-38.6	-44.2	5.6

Table S2. Dataset of 30 FXR ligands obtained from literature for testing the LIE models for the benzimidazole, isoxazole and sulfonamide classes of compounds, and of 60 FXR ligands of the D3R GC2 ligand set. Listed are the ligand ID used throughout the current work; ChEMBL or D3R (FXR) IDs of the ligands; literature references (Ref.) from which IC50 values were retrieved to obtain the experimentally observed FXR binding free energy ΔG_{obs} (in kJ mol^{-1}), calculated as ΔG_{obs} = RT ln (IC_{50}/2); the predicted binding free energy (ΔG_{pred}) in kJ mol^{-1}; differences $\Delta G_{obs} - \Delta G_{pred}$ (Diff., in kJ mol^{-1}); the violations for each of the 5 AD criteria (1 for violated and 0 for non-violated); and the total CI score per prediction.

Compound class	ID	ChEMBL/FXR ID	Ref.	ΔG_{obs}	ΔG_{pred}	Diff.	AD criteria					CI score
**Literature test**							1	2	3	4	5	
Benzimidazole	1	CHEMBL1642355	[5]	-33.8	-33.8	0.0	0	0	0	0	0	0
	2	CHEMBL1642360	[5]	-31.9	-43.1	11.2	0	0	0	0	1	1
	3	CHEMBL1642361	[5]	-31.7	-38.6	6.9	0	0	0	1	0	1
	4	CHEMBL1642351	[5]	-32.9	-37.5	4.5	0	0	0	0	0	0
	5	CHEMBL1642353	[5]	-28.2	-37.0	8.8	0	0	0	0	0	0
	6	CHEMBL1642357	[5]	-33.0	-38.9	6.0	0	0	0	0	0	0
	7	CHEMBL1642349	[5]	-27.7	-36.1	8.4	0	0	0	1	0	1
	8	CHEMBL1642348	[5]	-30.0	-38.5	8.6	0	1	0	1	0	2
	9	CHEMBL1642362	[15]	-47.1	-43.1	-4.0	0	0	0	0	0	0
	10	CHEMBL1668243	[15]	-48.1	-44.2	-3.8	0	0	0	1	0	1
	11	CHEMBL1668241	[15]	-39.1	-37.2	-2.0	0	0	0	0	0	0
	12	CHEMBL1668246	[15]	-35.4	-38.8	3.4	0	0	0	1	1	2
	13	CHEMBL1668255	[15]	-40.3	-37.0	-3.3	0	0	0	1	1	2
	14	CHEMBL1668251	[15]	-37.5	-37.9	0.4	0	0	0	1	0	1
	15	CHEMBL1668239	[15]	-44.3	-40.5	-3.8	0	0	0	0	0	0

	16	CHEMBL1668238	[15]	-47.8	-42.0	-5.8	0	0	0	0	0	0
	17	CHEMBL1668250	[15]	-32.9	-39.2	6.3	0	0	0	1	1	2
	18	CHEMBL1615150	[15]	-46.1	-36.5	-9.5	0	0	0	1	1	2
	19	CHEMBL1668257	[15]	-48.3	-45.1	-3.2	0	0	0	1	1	2
	20	CHEMBL1615152	[15]	-44.5	-40.1	-4.5	0	0	0	1	0	1
	21	CHEMBL1668249	[15]	-53.5	-44.2	-9.4	0	0	0	1	1	2
	22	CHEMBL1615153	[15]	-43.8	-41.5	-2.2	0	0	0	1	1	2
	23	CHEMBL1668252	[15]	-36.7	-36.6	-0.1	0	0	0	1	1	2
Sulfonamide	1	CHEMBL3264637	[17]	-46.9	-45.4	-1.5	0	0	0	1	0	1
	2	CHEMBL3264638	[17]	-45.6	-38.8	-6.8	0	0	1	1	1	3
	3	CHEMBL3264641	[17]	-43.6	-44.5	0.9	0	0	0	1	1	2
	4	CHEMBL3264643	[17]	-42.7	-43.5	0.9	0	0	0	1	0	1
	5	CHEMBL3264647	[17]	-40.3	-44.9	4.6	0	0	0	1	1	2
	6	CHEMBL3264649	[17]	-44.2	-43.7	-0.5	0	0	0	1	0	1
	7	CHEMBL3264988	[17]	-38.1	-43.8	5.8	0	0	0	1	1	2
**D3R predictions**												
Benzimidazole	1	FXR_19		-42.3	-37.5	-4.8	0	0	0	0	0	0
	2	FXR_27		-48.1	-36.3	-11.8	0	0	0	1	0	1
	3	FXR_62		-40.7	-41.9	1.1	0	0	0	1	1	2
	4	FXR_60		-47.0	-46.8	-0.2	0	1	0	1	1	3
	5	FXR_52		-40.7	-42.4	1.7	0	0	0	1	0	1
	6	FXR_20		-48.8	-40.8	-7.9	0	0	0	0	0	0
	7	FXR_50		-32.2	-36.7	4.5	0	0	0	0	0	0
	8	FXR_25		-45.0	-42.1	-2.9	0	0	0	0	0	0
	9	FXR_58		-46.3	-40.9	-5.5	0	0	0	1	1	2
	10	FXR_72		-47.4	-43.7	-3.7	0	0	0	1	1	2
	11	FXR_24		-46.0	-38.5	-7.5	0	0	0	1	1	2
	12	FXR_56		-30.5	-47.5	17.0	0	0	0	1	1	2
	13	FXR_53		-40.9	-38.1	-2.8	0	0	0	1	0	1

14	FXR_59	-39.3	-46.0	6.7	0	0	0	1	0	1
15	FXR_21	-37.0	-42.2	5.3	0	0	0	0	0	0
16	FXR_54	-36.8	-39.3	2.5	0	0	0	0	0	0
17	FXR_61	-47.5	-37.5	-10.0	0	1	0	1	0	2
18	FXR_28	-45.9	-40.7	-5.3	0	1	0	0	1	2
19	FXR_29	-44.4	-42.7	-1.7	0	1	0	1	0	2
20	FXR_71	-45.0	-43.5	-1.5	0	1	0	1	1	3
21	FXR_42	-45.8	-34.4	-11.3	0	0	0	1	0	1
22	FXR_32	-43.0	-33.6	-9.4	0	0	0	1	0	1
23	FXR_22	-49.8	-45.4	-4.5	0	0	0	1	0	1
24	FXR_64	-40.9	-32.7	-8.2	0	0	0	1	0	1
25	FXR_68	-26.3	-36.0	9.8	0	0	0	1	1	2
26	FXR_69	-34.6	-51.4	16.7	1	0	1	1	1	4
27	FXR_66	-30.4	-39.0	8.6	0	0	0	1	1	2
28	FXR_67	-29.3	-40.8	11.5	0	0	0	1	1	2
29	FXR_31	-50.0	-34.1	-15.9	0	0	0	1	0	1
30	FXR_57	-49.8	-35.8	-14.0	0	0	0	1	0	1
31	FXR_55	-58.3	-38.4	-19.9	0	0	0	1	0	1
32	FXR_26	-34.7	-41.9	7.2	0	0	0	1	0	1
33	FXR_70	-38.2	-40.2	2.0	0	0	0	1	0	1
34	FXR_36	-45.6	-43.2	-2.4	0	0	0	1	0	1
35	FXR_63	-27.9	-43.5	15.6	0	0	0	1	1	2
36	FXR_51	-41.5	-36.4	-5.0	0	0	0	1	0	1
37	FXR_30	-42.8	-41.3	-1.5	0	0	0	1	1	2
38	FXR_37	-30.0	-35.0	5.0	0	0	0	0	0	0
39	FXR_6	-45.1	-35.5	-9.6	0	0	0	1	1	2
40	FXR_9	-33.4	-37.3	3.8	0	0	0	1	0	1
41	FXR_13	-47.3	-49.6	2.3	0	0	0	1	1	2
42	FXR_39	-43.4	-36.4	-7.1	0	0	0	0	0	0

	43	FXR_40	-32.5	-43.2	10.7	0	0	0	1	0	1
	44	FXR_8	-33.9	-42.1	8.2	0	0	0	1	0	1
	45	FXR_7	-41.6	-43.1	1.5	0	0	0	0	0	0
	46	FXR_14	-47.9	-37.4	-10.5	0	0	0	1	0	1
Isoxazole	1	FXR_4	-31.3	-35.3	3.9	0	1	1	1	1	4
	2	FXR_23	-37.7	-36.2	-1.5	0	1	0	1	1	3
	3	FXR_33	-44.0	-39.2	-4.9	0	0	0	1	0	1
	4	FXR_65	-42.2	-37.2	-5.0	0	0	0	1	0	1
Sulfonamide	1	FXR_90	-24.8	-43.2	18.4	0	1	0	1	0	2
	2	FXR_102	-27.8	-43.6	15.8	0	0	0	1	1	2
	3	FXR_15	-28.0	-43.5	15.4	0	1	0	0	1	2
Spiro	1	FXR_81	-33.8	-42.0	8.3	0	1	0	1	0	2
Miscellaneous	1	FXR_1	-26.2	-41.5	15.3	0	1	0	1	0	2
	2	FXR_2	-36.6	-36.6	0.0	0	1	1	1	1	4
	3	FXR_3	-22.3	-34.3	12.0	0	1	1	1	1	4
	4	FXR_5	-35.4	-37.6	2.2	0	1	0	1	0	2
	5	FXR_18	-33.4	-33.0	-0.4	0	1	0	1	1	3
	6	FXR_34	-48.4	-37.0	-11.4	0	1	0	0	1	2

Figure S1: Molecular structures of literature training compounds

	Compound class	ID	ChEMBL ID	Structure
**1**	Benzimidazole	1	CHEMBL1642356	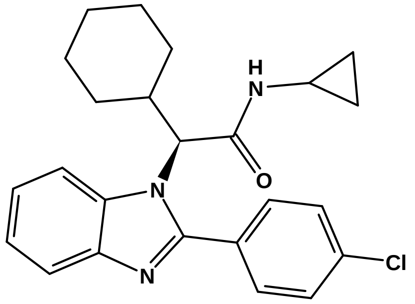
**2**	Benzimidazole	2	CHEMBL1642359	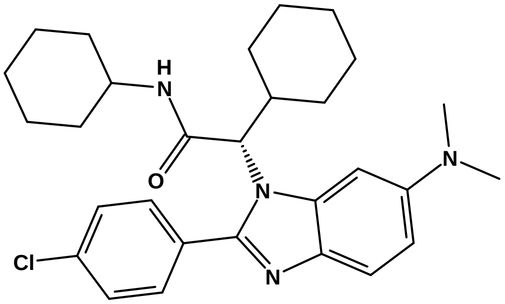
**3**	Benzimidazole	3	CHEMBL1642358	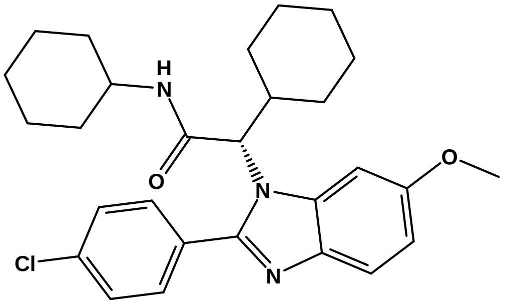
**4**	Benzimidazole	4	CHEMBL1642346	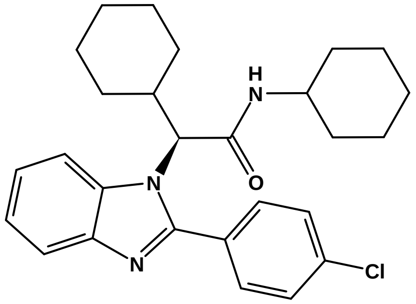
**5**	Benzimidazole	5	CHEMBL1642350	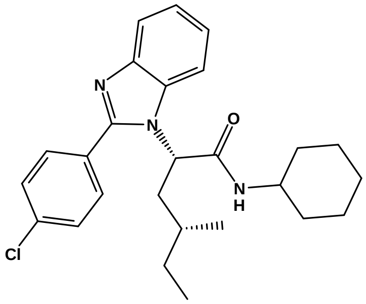

	Compound class	ID	ChEMBL ID	Structure
**6**	Benzimidazole	6	CHEMBL1642354	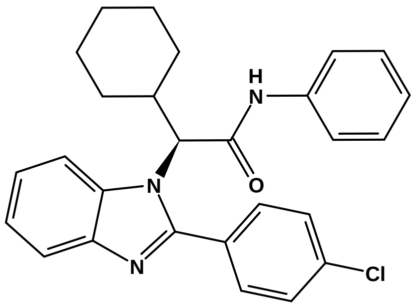
**7**	Benzimidazole	7	CHEMBL1642347	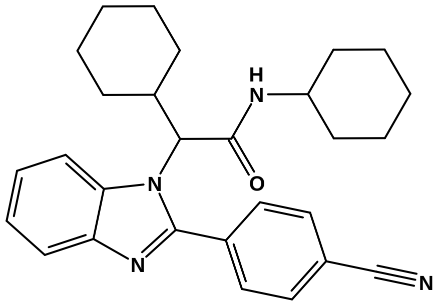
**8**	Benzimidazole	8	CHEMBL1668262	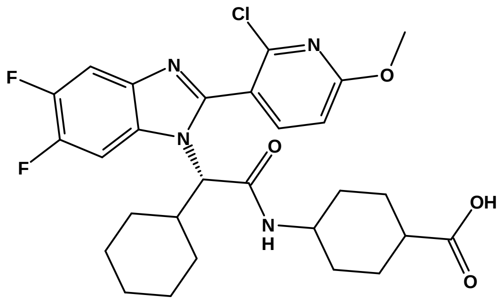
**9**	Benzimidazole	9	CHEMBL1668242	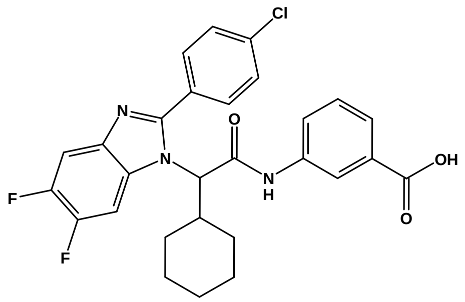
**10**	Benzimidazole	10	CHEMBL1668247	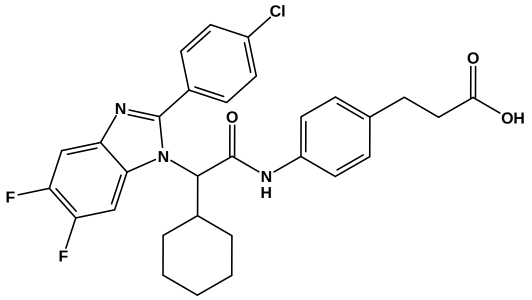

	Compound class	ID	ChEMBL ID	Structure
**11**	Benzimidazole	11	CHEMBL1668256	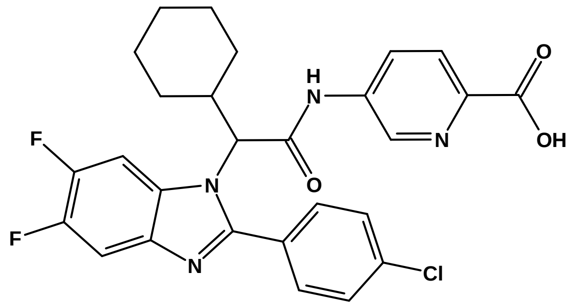
**12**	Benzimidazole	12	CHEMBL1668254	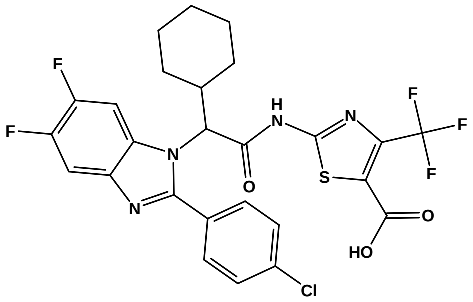
**13**	Benzimidazole	13	CHEMBL1668253	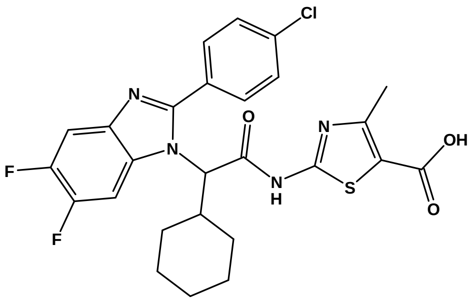
**14**	Benzimidazole	14	CHEMBL1668240	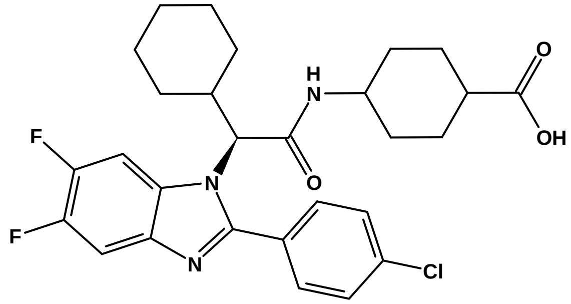
**15**	Benzimidazole	15	CHEMBL1668244	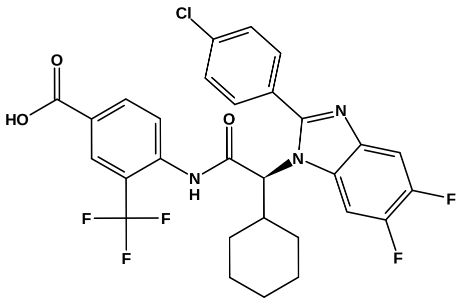

	Compound class	ID	ChEMBL ID	Structure
**16**	Benzimidazole	16	CHEMBL1668259	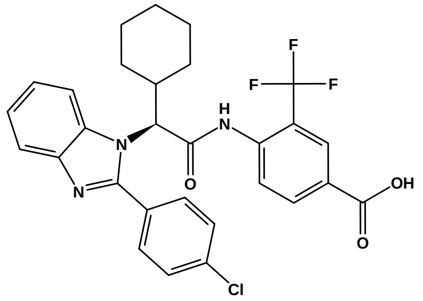
**17**	Benzimidazole	17	CHEMBL1668258	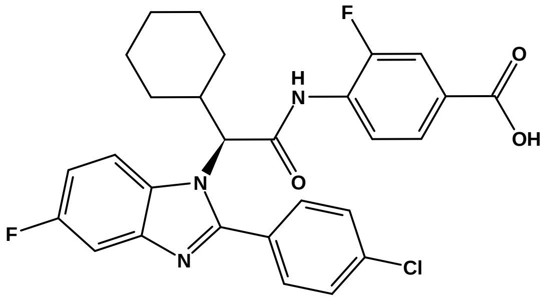
**18**	Benzimidazole	18	CHEMBL1615154	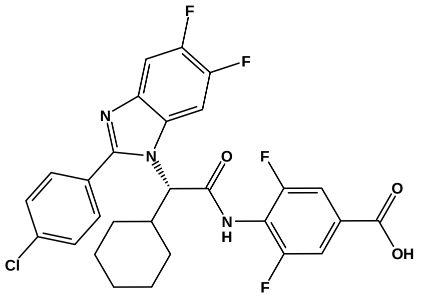
**19**	Benzimidazole	19	CHEMBL1668248	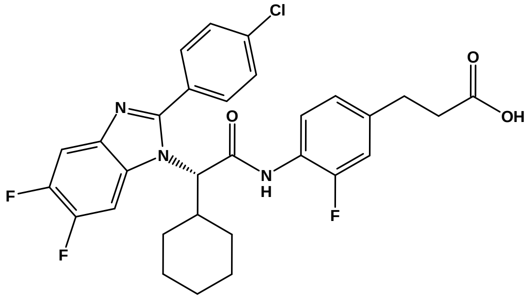
**20**	Benzimidazole	20	CHEMBL1668245	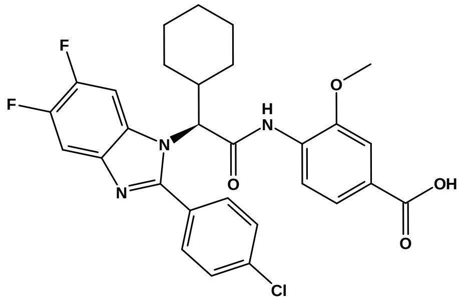

	Compound class	ID	ChEMBL ID	Structure
**21**	Benzimidazole	21	CHEMBL1668260	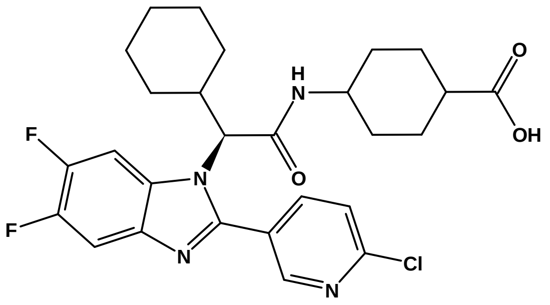
**22**	Benzimidazole	22	CHEMBL1668261	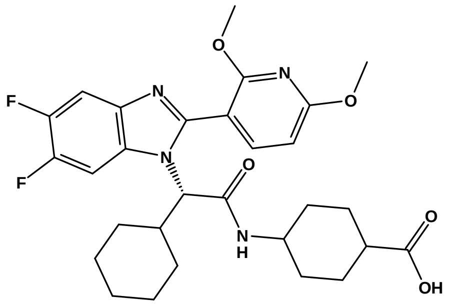
**23**	Isoxazole	1	CHEMBL511389	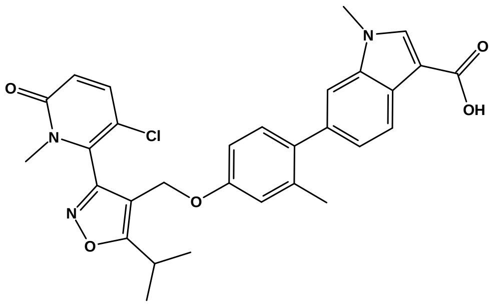
**24**	Isoxazole	2	CHEMBL511559	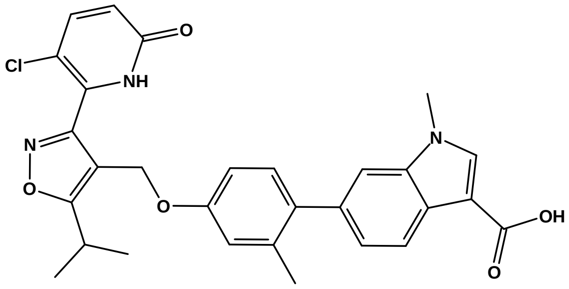
**25**	Isoxazole	3	CHEMBL466880	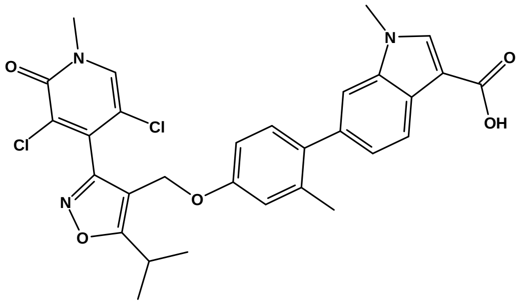

	Compound class	ID	ChEMBL ID	Structure
**26**	Isoxazole	4	CHEMBL466471	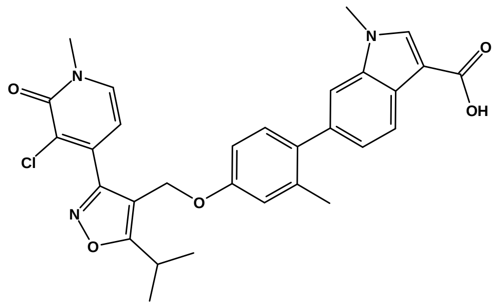
**27**	Isoxazole	5	CHEMBL513149	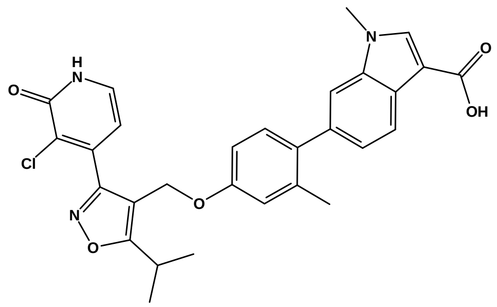
**28**	Isoxazole	6	CHEMBL466523	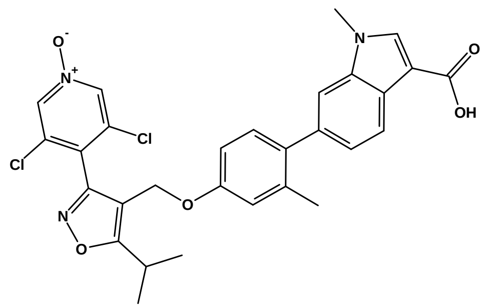
**29**	Isoxazole	7	CHEMBL466323	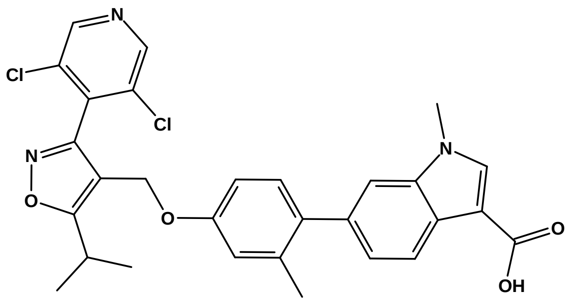
**30**	Isoxazole	8	CHEMBL466887	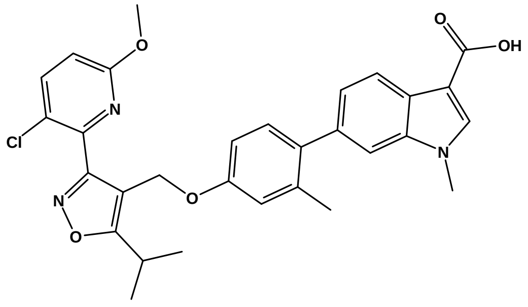

	Compound class	ID	ChEMBL ID	Structure
31	Isoxazole	9	CHEMBL466318	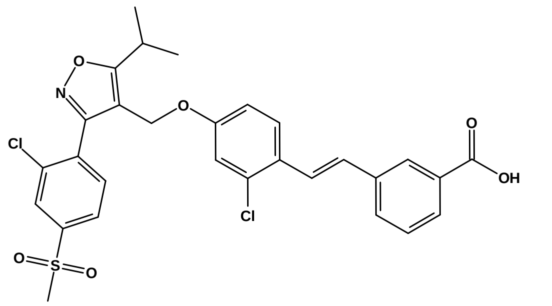
32	Isoxazole	10	CHEMBL469135	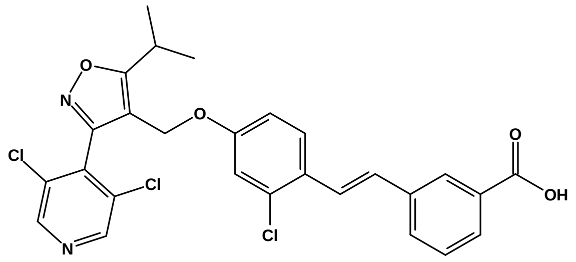
33	Isoxazole	11	CHEMBL466315	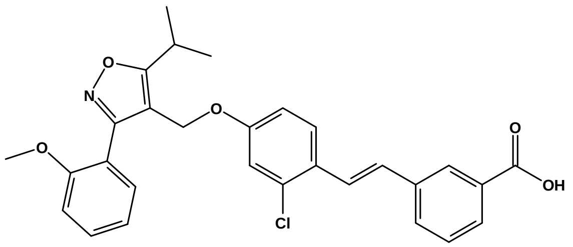
34	Sulfonamide	1	CHEMBL1367639	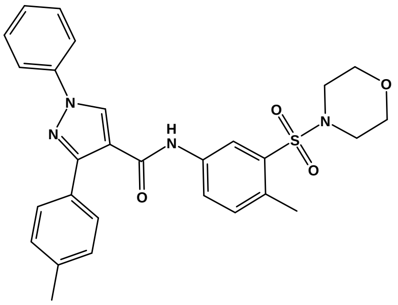
35	Sulfonamide	2	CHEMBL3264627	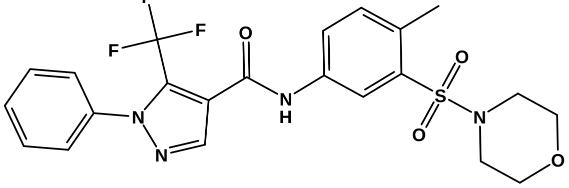

	Compound class	ID	ChEMBL ID	Structure
**36**	Sulfonamide	3	CHEMBL3264635	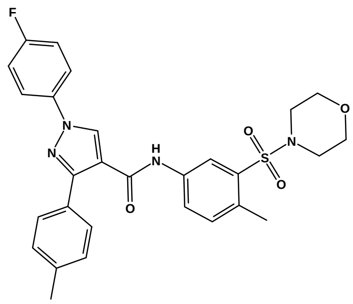
**37**	Sulfonamide	4	CHEMBL3264639	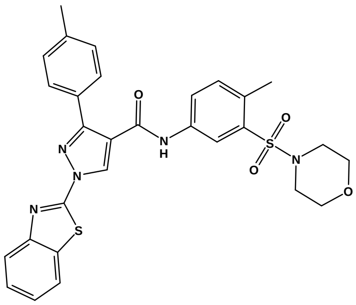
**38**	Sulfonamide	5	CHEMBL3264640	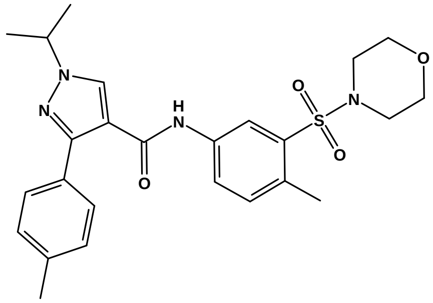
**39**	Sulfonamide	6	CHEMBL3264642	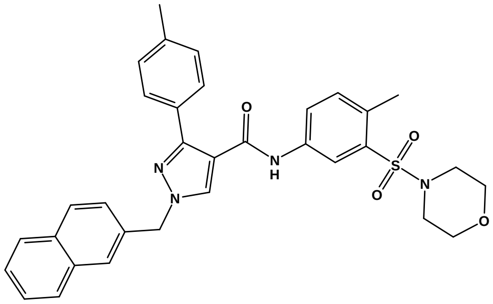
**40**	Sulfonamide	7	CHEMBL3264644	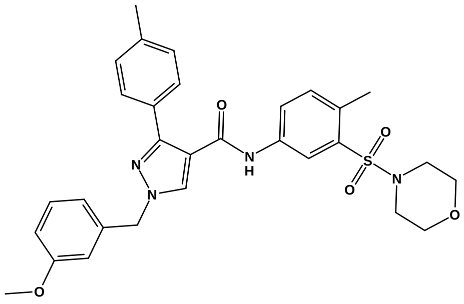

	Compound class	ID	ChEMBL ID	Structure
**41**	Sulfonamide	8	CHEMBL3264645	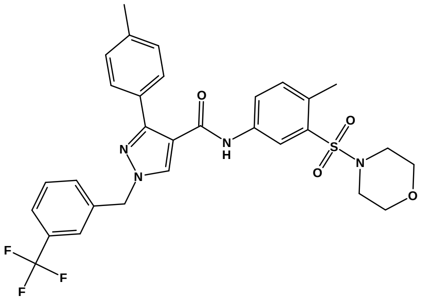
**42**	Sulfonamide	9	CHEMBL3264646	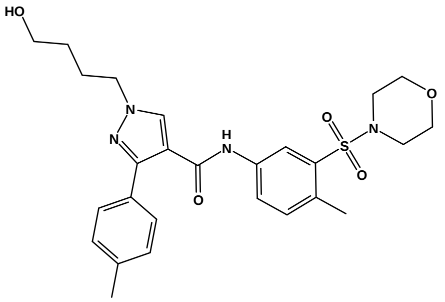
**43**	Sulfonamide	10	CHEMBL3264648	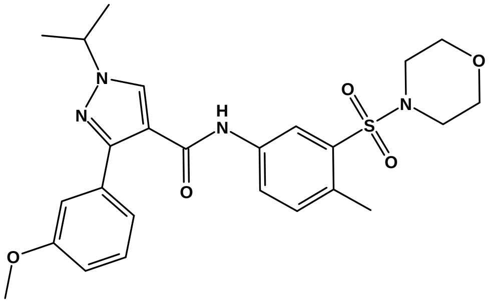
**44**	Sulfonamide	11	CHEMBL3264650	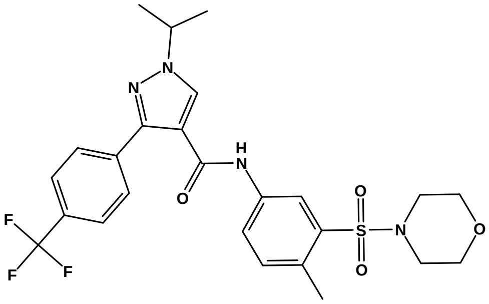
**45**	Sulfonamide	12	CHEMBL3264651	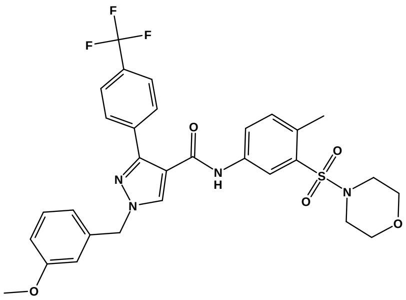

	Compound class	ID	ChEMBL ID	Structure
**46**	Sulfonamide	13	CHEMBL3264987	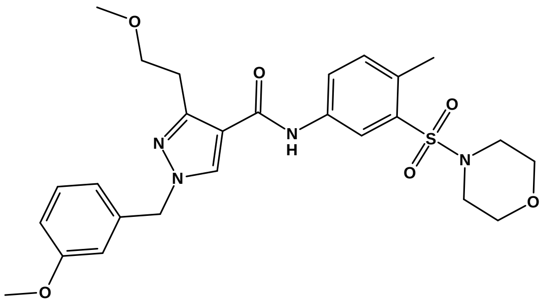

Figure S2: Molecular structures of literature test compounds

	Compound class	ID	ChEMBLID	Structure
**1**	Benzimidazole	1	CHEMBL1642355	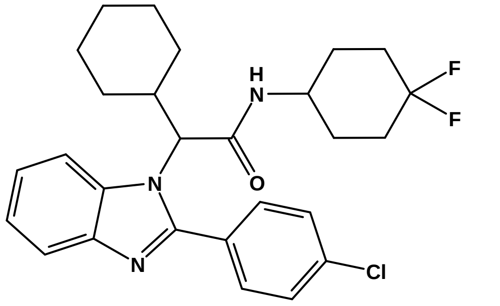
**2**	Benzimidazole	2	CHEMBL1642360	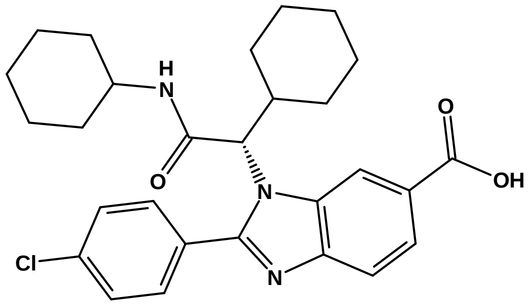
**3**	Benzimidazole	3	CHEMBL1642361	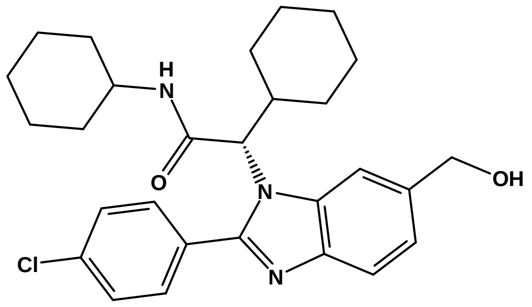
**4**	Benzimidazole	4	CHEMBL1642351	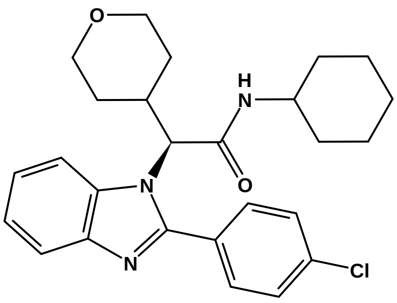
**5**	Benzimidazole	5	CHEMBL1642353	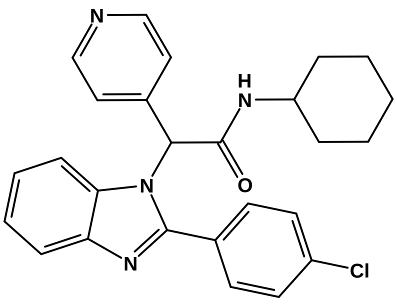

	Compound class	ID	ChEMBLID	Structure
**6**	Benzimidazole	6	CHEMBL1642357	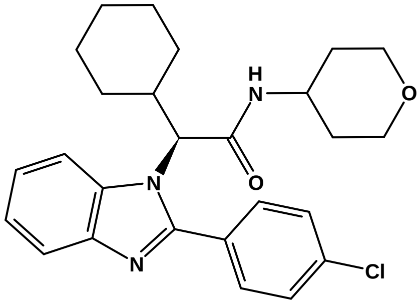
**7**	Benzimidazole	7	CHEMBL1642349	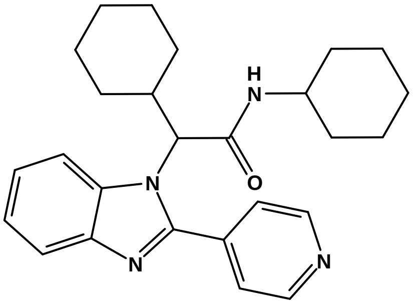
**8**	Benzimidazole	8	CHEMBL1642348	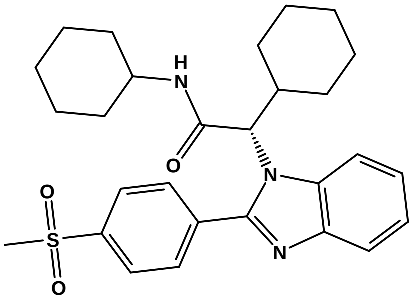
**9**	Benzimidazole	9	CHEMBL1642362	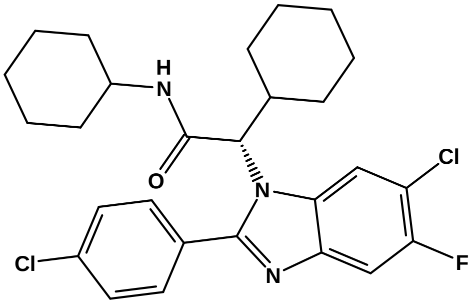
**10**	Benzimidazole	10	CHEMBL1668243	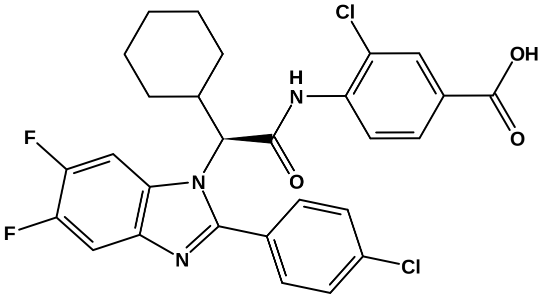

	Compound class	ID	ChEMBLID	Structure
11	Benzimidazole	11	CHEMBL1668241	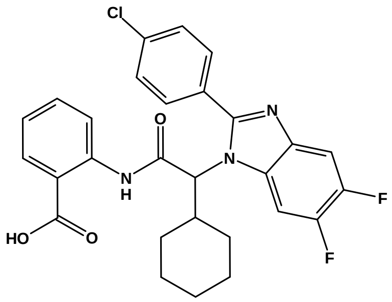
12	Benzimidazole	12	CHEMBL1668246	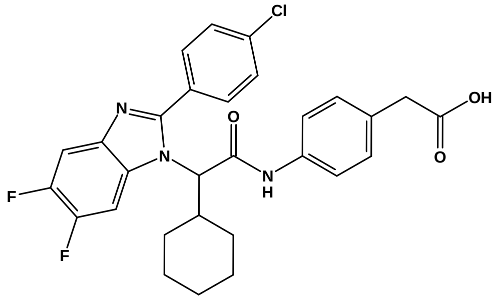
13	Benzimidazole	13	CHEMBL1668255	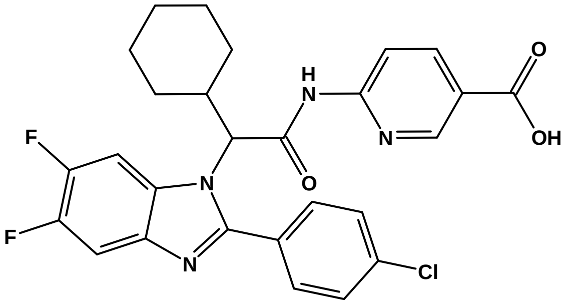
14	Benzimidazole	14	CHEMBL1668251	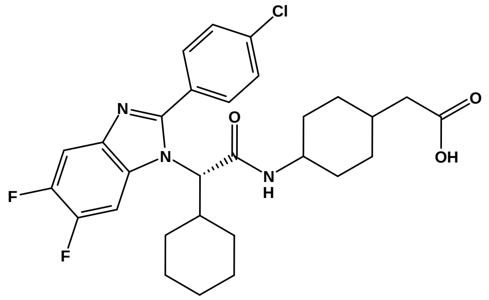
15	Benzimidazole	15	CHEMBL1668239	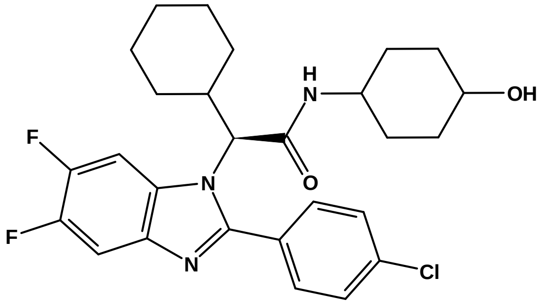

	Compound class	ID	ChEMBLID	Structure
**16**	Benzimidazole	16	CHEMBL1668238	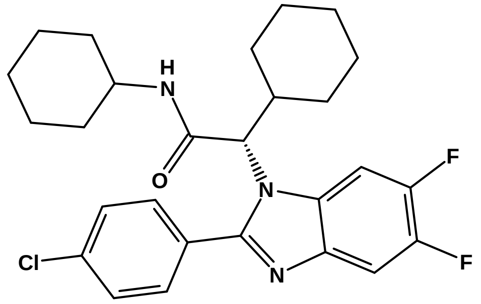
**17**	Benzimidazole	17	CHEMBL1668250	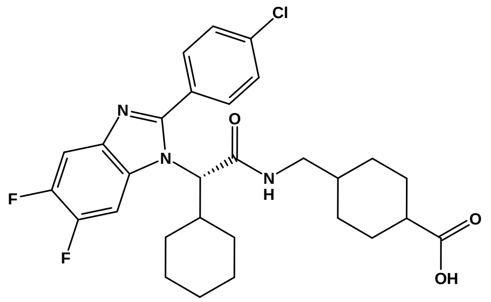
**18**	Benzimidazole	18	CHEMBL1615150	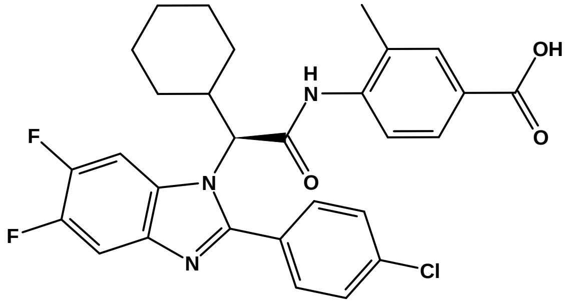
**19**	Benzimidazole	19	CHEMBL1668257	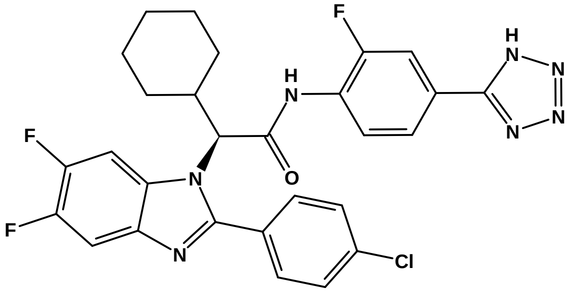
**20**	Benzimidazole	20	CHEMBL1615152	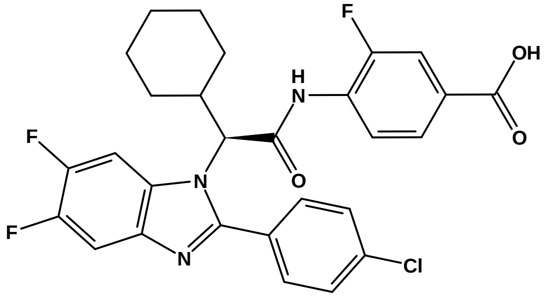

	Compound class	ID	ChEMBLID	Structure
**21**	Benzimidazole	21	CHEMBL1668249	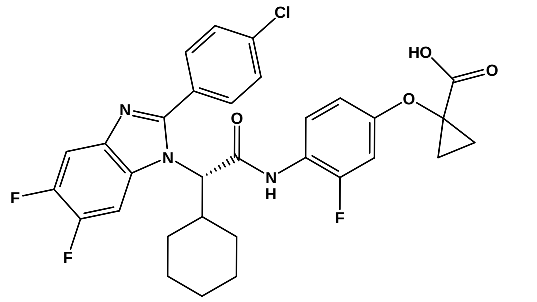
**22**	Benzimidazole	22	CHEMBL1615153	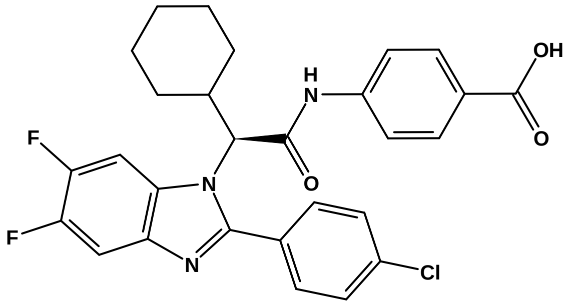
**23**	Benzimidazole	23	CHEMBL1668252	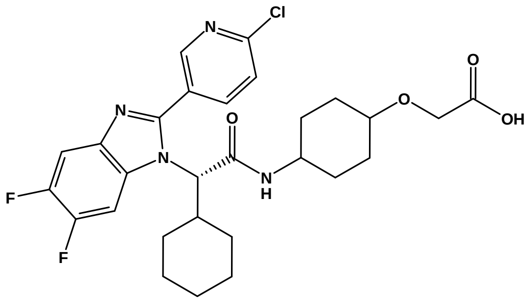
**24**	Sulfonamide	1	CHEMBL3264637	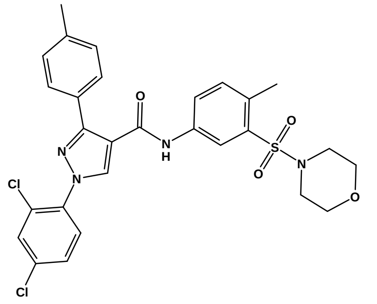
**25**	Sulfonamide	2	CHEMBL3264638	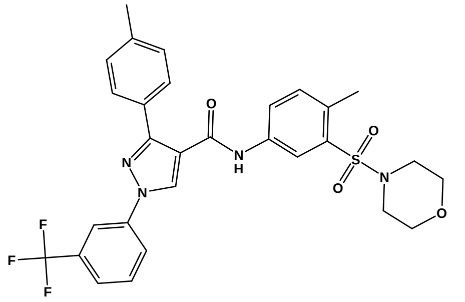

	Compound class	ID	ChEMBLID	Structure
**26**	Sulfonamide	3	CHEMBL3264641	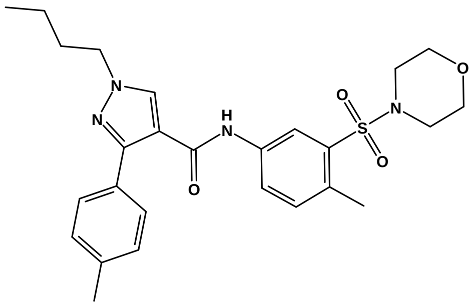
**27**	Sulfonamide	4	CHEMBL3264643	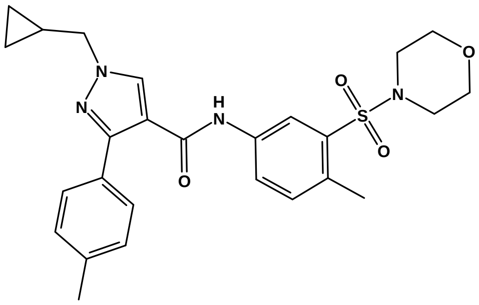
**28**	Sulfonamide	5	CHEMBL3264647	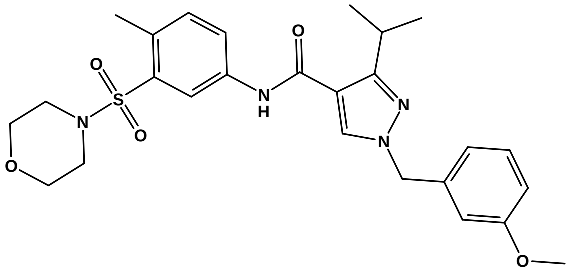
**29**	Sulfonamide	6	CHEMBL3264649	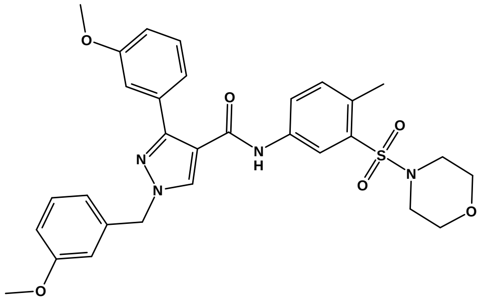
**30**	Sulfonamide	7	CHEMBL3264988	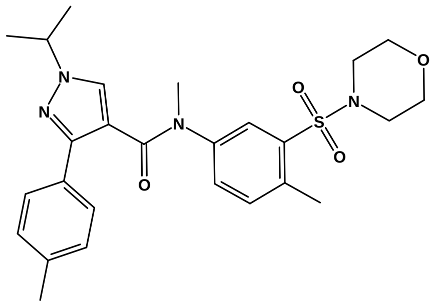

Figure S3: Molecular structures of D3R compounds

	Compound class	ID	FXR ID	Structure
**1**	Benzimidazole	1	FXR_19	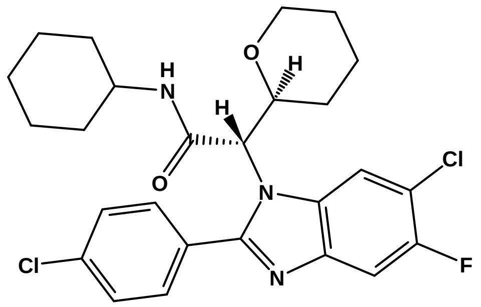
**2**	Benzimidazole	2	FXR_27	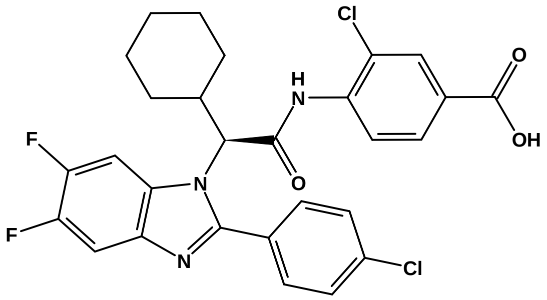
**3**	Benzimidazole	3	FXR_62	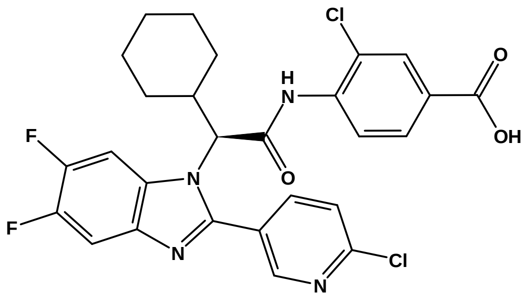
**4**	Benzimidazole	4	FXR_60	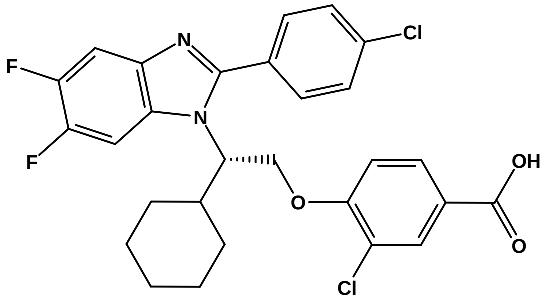
**5**	Benzimidazole	5	FXR_52	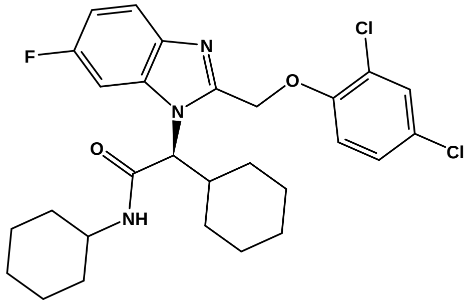

	Compound class	ID	FXR ID	Structure
**6**	Benzimidazole	6	FXR_20	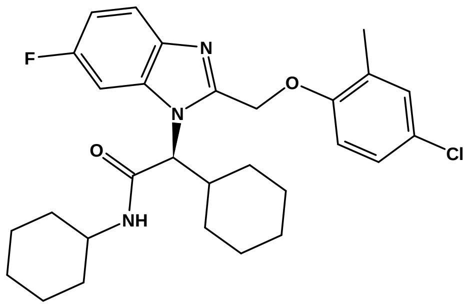
**7**	Benzimidazole	7	FXR_50	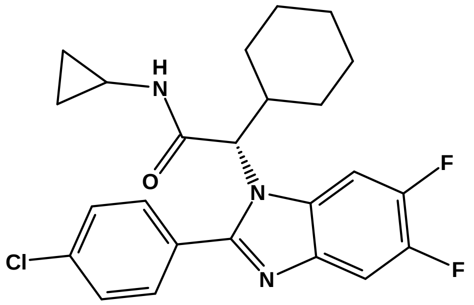
**8**	Benzimidazole	8	FXR_25	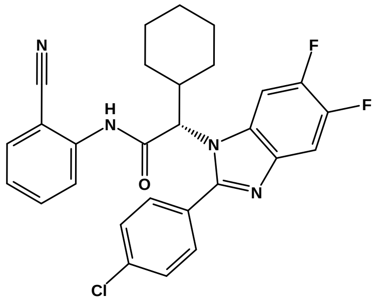
**9**	Benzimidazole	9	FXR_58	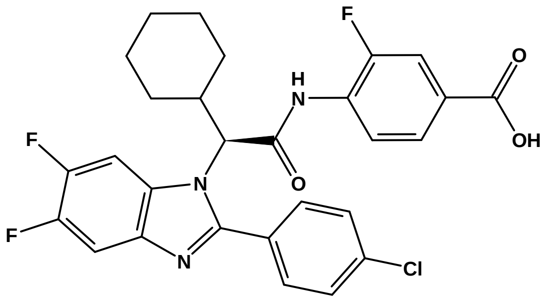
**10**	Benzimidazole	10	FXR_72	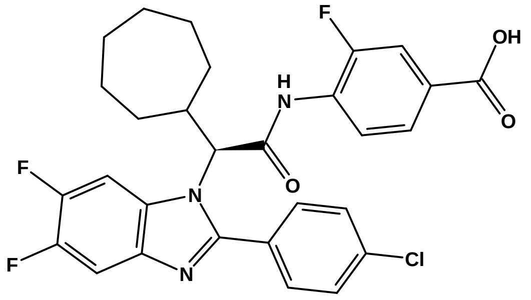

	Compound class	ID	FXR ID	Structure
**11**	Benzimidazole	11	FXR_24	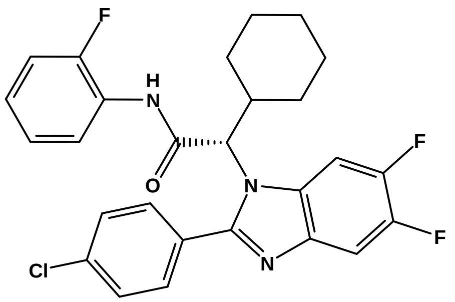
**12**	Benzimidazole	12	FXR_56	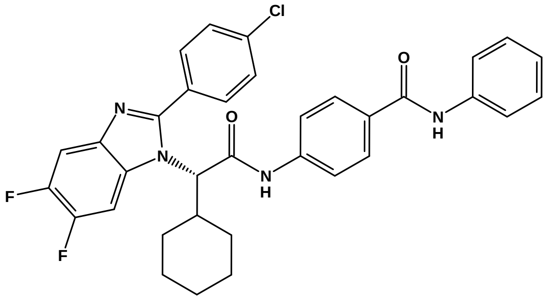
**13**	Benzimidazole	13	FXR_53	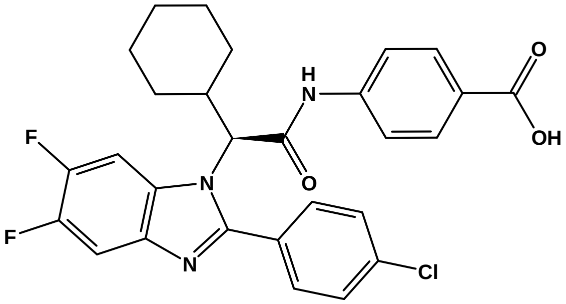
**14**	Benzimidazole	14	FXR_59	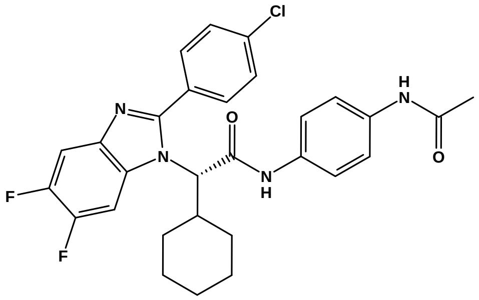
**15**	Benzimidazole	15	FXR_21	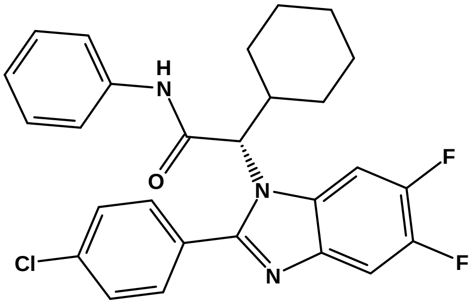

	Compound class	ID	FXR ID	Structure
**16**	Benzimidazole	16	FXR_54	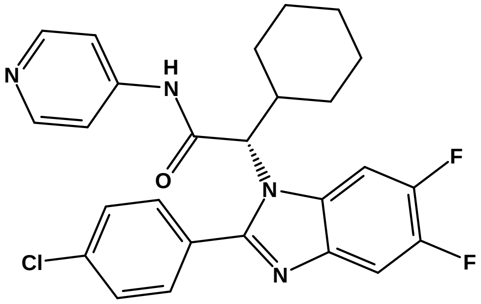
**17**	Benzimidazole	17	FXR_61	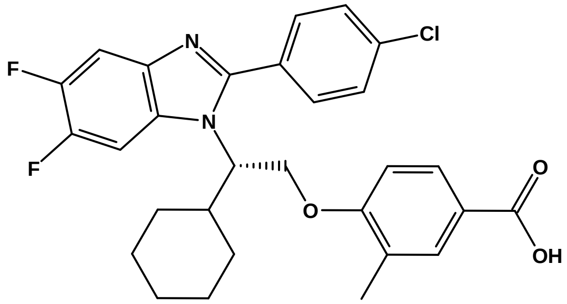
**18**	Benzimidazole	18	FXR_28	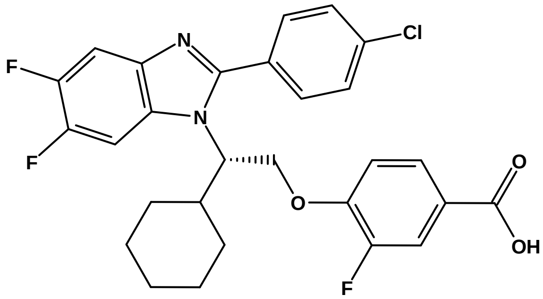
**19**	Benzimidazole	19	FXR_29	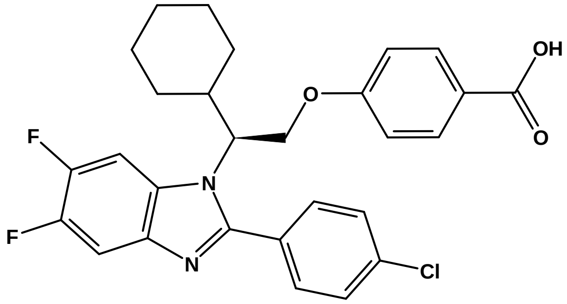
**20**	Benzimidazole	20	FXR_71	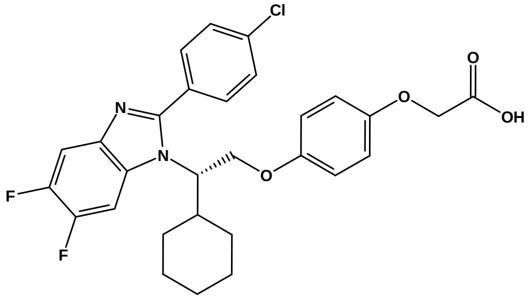

	Compound class	ID	FXR ID	Structure
**21**	Benzimidazole	21	FXR_42	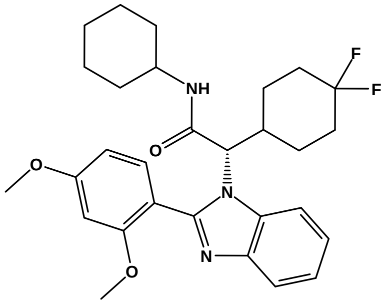
**22**	Benzimidazole	22	FXR_32	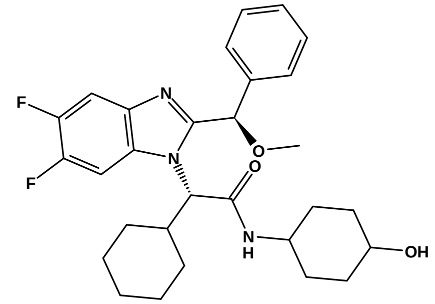
**23**	Benzimidazole	23	FXR_22	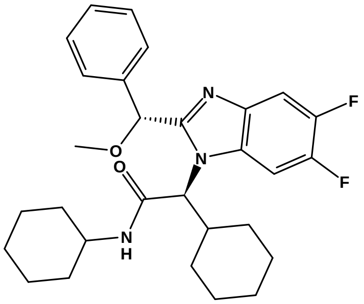
**24**	Benzimidazole	24	FXR_64	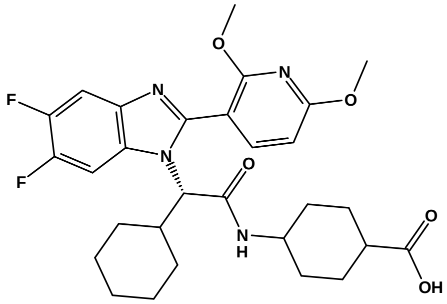
**25**	Benzimidazole	25	FXR_68	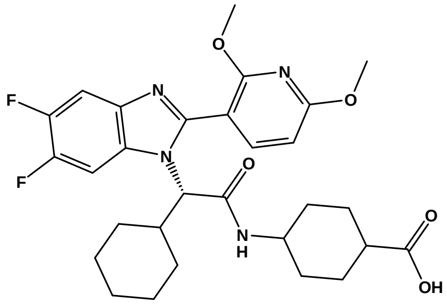

	Compound class	ID	FXR ID	Structure
**26**	Benzimidazole	26	FXR_69	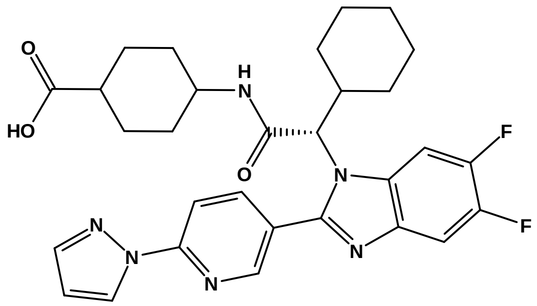
**27**	Benzimidazole	27	FXR_66	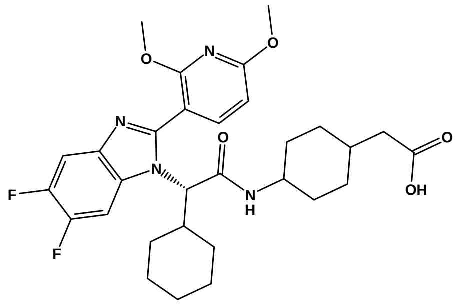
**28**	Benzimidazole	28	FXR_67	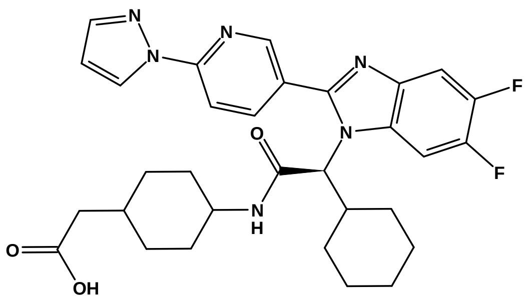
**29**	Benzimidazole	29	FXR_31	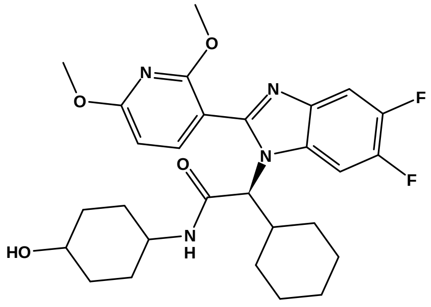
**30**	Benzimidazole	30	FXR_57	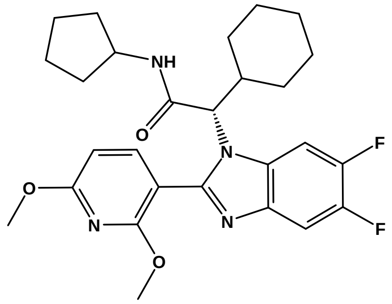

	Compound class	ID	FXR ID	Structure
**31**	Benzimidazole	31	FXR_55	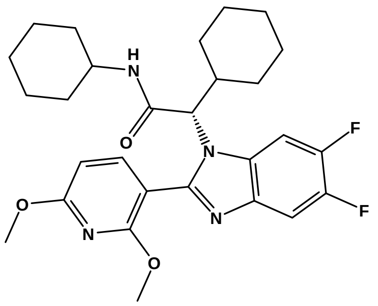
**32**	Benzimidazole	32	FXR_26	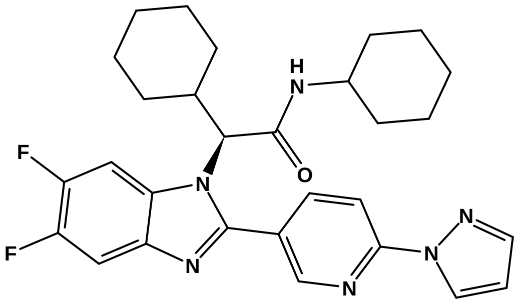
**33**	Benzimidazole	33	FXR_70	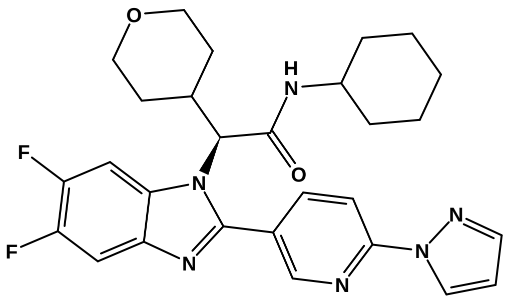
**34**	Benzimidazole	34	FXR_36	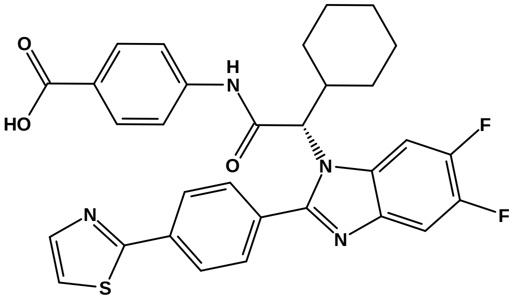
**35**	Benzimidazole	35	FXR_63	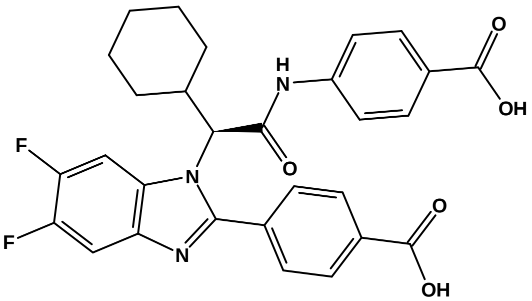

	Compound class	ID	FXR ID	Structure
**36**	Benzimidazole	36	FXR_51	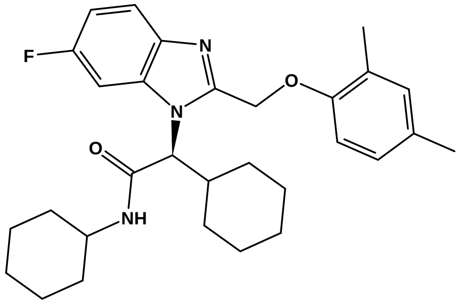
**37**	Benzimidazole	37	FXR_30	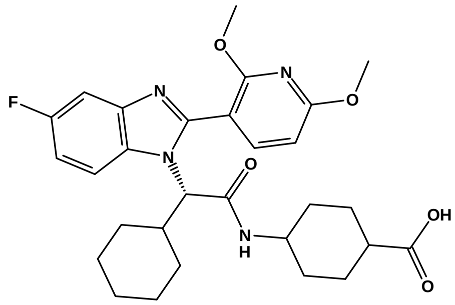
**38**	Benzimidazole	38	FXR_37	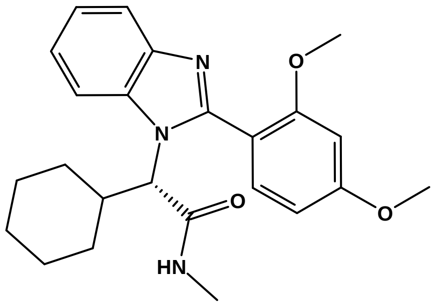
**39**	Benzimidazole	39	FXR_6	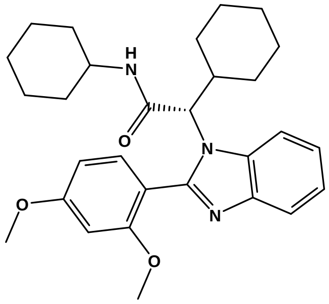
**40**	Benzimidazole	40	FXR_9	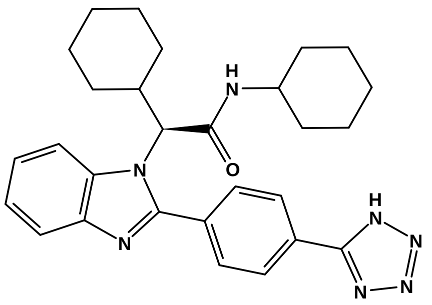

	Compound class	ID	FXR ID	Structure
**41**	Benzimidazole	41	FXR_13	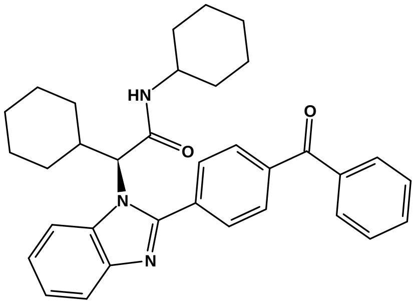
**42**	Benzimidazole	42	FXR_39	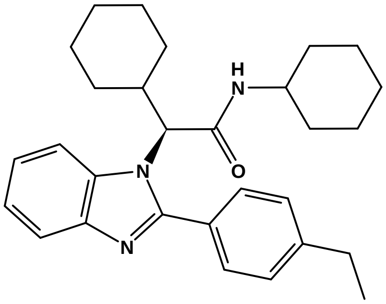
**43**	Benzimidazole	43	FXR_40	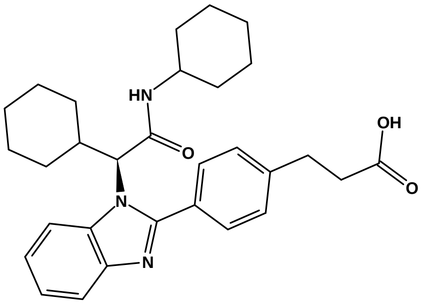
**44**	Benzimidazole	44	FXR_8	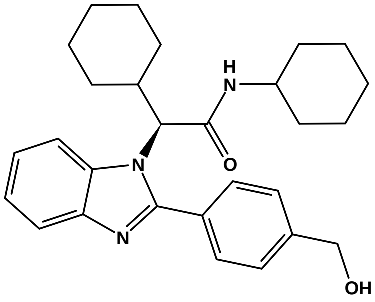
**45**	Benzimidazole	45	FXR_7	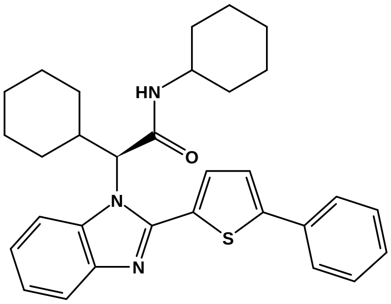

	Compound class	ID	FXR ID	Structure
**46**	Benzimidazole	46	FXR_14	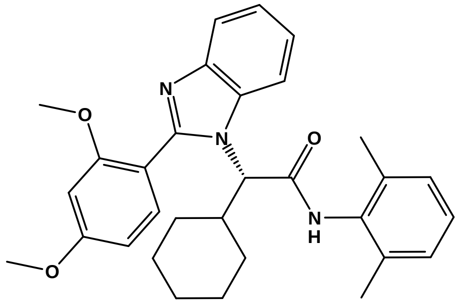
**47**	Isoxazole	1	FXR_4	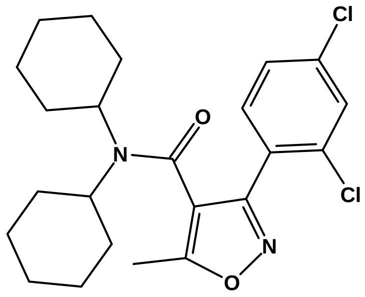
**48**	Isoxazole	2	FXR_23	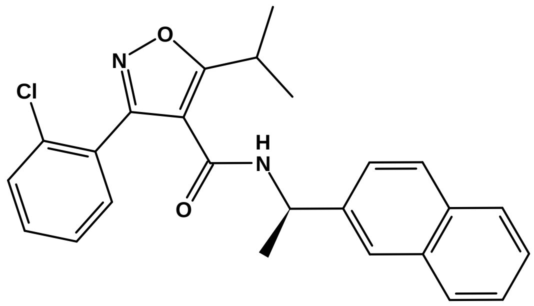
**49**	Isoxazole	3	FXR_33	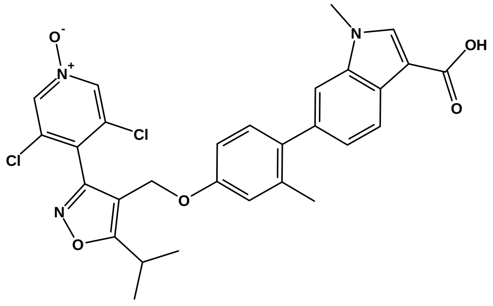
**50**	Isoxazole	4	FXR_65	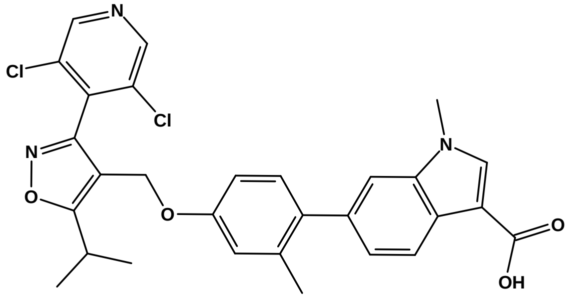

	Compound class	ID	FXR ID	Structure
**51**	Sulfonamide	1	FXR_90	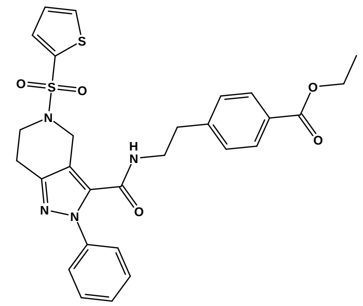
**52**	Sulfonamide	2	FXR_102	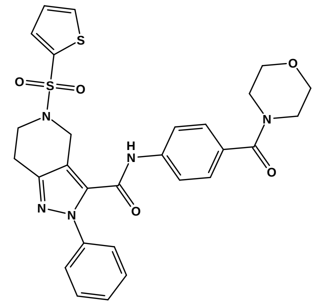
**53**	Sulfonamide	3	FXR_15	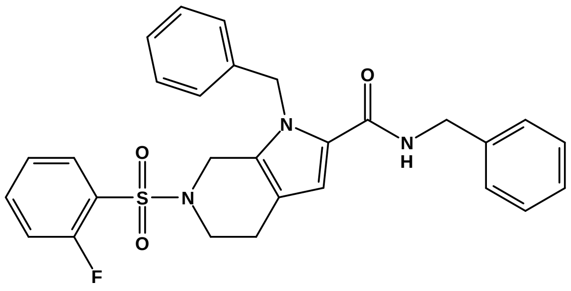
**54**	Spiro	1	FXR_81	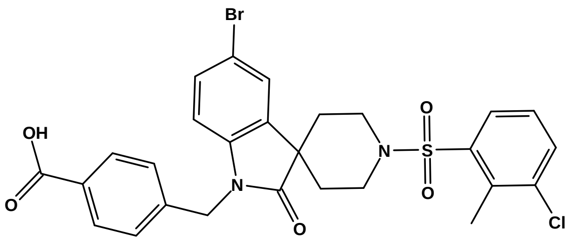
**55**	Miscellaneous	1	FXR_1	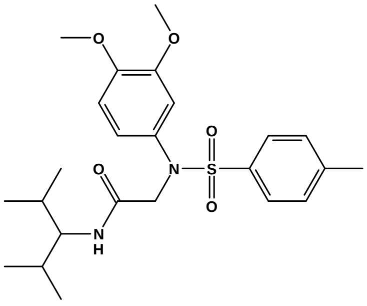

	Compound class	ID	FXR ID	Structure
**56**	Miscellaneous	2	FXR_2	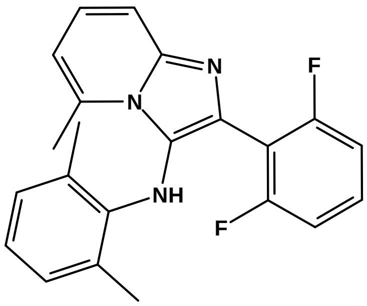
**57**	Miscellaneous	3	FXR_3	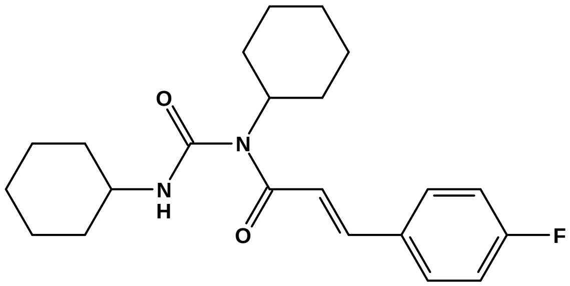
**58**	Miscellaneous	4	FXR_5	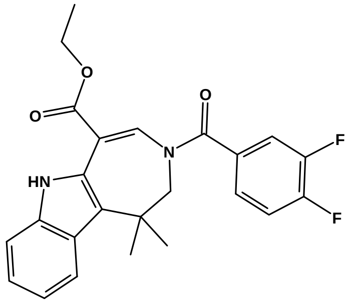
**59**	Miscellaneous	5	FXR_18	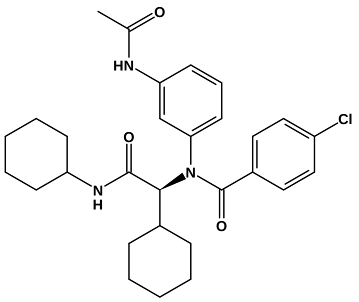
**60**	Miscellaneous	6	FXR_34	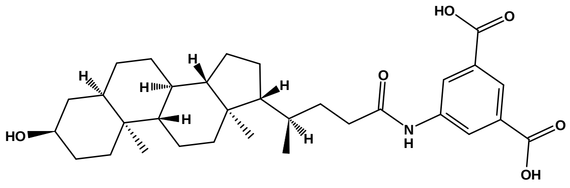
